# The dose-response relationship of subretinal gene therapy with rAAV2tYF-CB-h*RS1* in a mouse model of X-linked retinoschisis

**DOI:** 10.3389/fmed.2024.1304819

**Published:** 2024-02-13

**Authors:** Salma Hassan, Ying Hsu, Jacob M. Thompson, Emily Kalmanek, Joel A. VandeLune, Sarah Stanley, Arlene V. Drack

**Affiliations:** ^1^Department of Ophthalmology and Visual Sciences, Institute for Vision Research, and Carver College of Medicine, University of Iowa, Iowa City, IA, United States; ^2^Biomedical Science—Cell and Developmental Biology Graduate Program, Department of Anatomy and Cell Biology, University of Iowa, Iowa City, IA, United States; ^3^Department of Epidemiology, College of Public Health, University of Iowa, Iowa City, IA, United States; ^4^Department of Pediatrics, University of Iowa, Iowa City, IA, United States

**Keywords:** X-linked retinoschisis, subretinal gene therapy, dose-response, *Rs1* knockout mouse, electroretinogram, visually guided swim assay, functional vision

## Abstract

**Purpose:**

X-linked retinoschisis (XLRS), due to loss-of-function mutations in the retinoschisin (*RS1*) gene, is characterized by a modest to severe decrease in visual acuity. Clinical trials for XLRS utilizing intravitreal (IVT) gene therapy showed ocular inflammation. We conducted a subretinal dose–response preclinical study using rAAV2tYF-CB-h*RS1* utilizing the *Rs1* knockout (*Rs1*-KO) mouse to investigate short- and long-term retinal rescue after subretinal gene delivery.

**Methods:**

*Rs1*-KO mice were subretinally injected with 2 μL of rAAV2tYF-CB-h*RS1* vector with 8E9 viral genomes (vg)/eye, 8E8 vg/eye, 8E7 vg/eye, or sham injection, and compared to untreated eyes. Reconstitution of human RS1 protein was detected using western blotting. Analysis of retinal function by electroretinography (ERG) and structural analysis by optical coherence tomography (OCT) were performed at 1, 2, 3, 5, 7, and 12 months post injection (MPI). Immunohistochemistry (IHC) was performed to evaluate cone rescue on the cellular level. Functional vision was evaluated using a visually guided swim assay (VGSA).

**Results:**

Western blotting analysis showed human RS1 protein expression in a dose-dependent manner. Quantification of western blotting showed that the RS1 protein expression in mice treated with the 8E8 vg dose was near the wild-type (WT) expression levels. ERG demonstrated dose-dependent effects: At 1 MPI the 8E8 vg dose treated eyes had higher light-adapted (LA) ERG amplitudes in 3.0 flash and 5 Hz flicker compared to untreated (*p* < 0.0001) and sham-treated eyes (*p* < 0.0001) which persisted until the 12 MPI endpoint, consistent with improved cone function. ERG b-wave amplitudes were higher in response to dark-adapted (DA) 0.01 dim flash and 3.0 standard combined response (SCR) compared to sham-treated (*p* < 0.01) and untreated eyes (*p* < 0.001) which persisted until 3 MPI, suggesting short-term improvement of the rod photoreceptors. All injections, including sham-treated, resulted in a cyst severity score of 1 (no cavities), with significant reductions compared to untreated eyes up to 3 MPI (*p* < 0.05). The high and low dose groups showed inconsistent ERG improvements, despite reduced cyst severity, emphasizing the dose-dependent nature of gene augmentation’s efficacy and the tenuous connection between cyst reduction and ERG improvement. IHC data showed a significant cone rescue in eyes treated with the 8E8 vg dose compared to sham-treated and untreated eyes. VGSA showed better functional vision in 8E8 vg dose treated mice. Eyes treated with the highest dose showed occasional localized degeneration in the outer nuclear layer.

**Conclusion:**

Our data suggest that a dose of 8E8 vg/eye subretinally improves retinal function and structure in the *Rs1*-KO mouse. It improves cone function, rod function, and reduces cyst severity. Sham treatment resolves schisis cysts, but 8E8 vg/eye is needed for optimal retinal electrical function rescue. These findings offer a promising path for clinical translation to human trials.

## 1 Introduction

X-linked retinoschisis (XLRS), also called juvenile X-linked retinoschisis, is a retinal disease that primarily affects males. It is due to loss-of-function mutations in the retinoschisin (*RS1*) gene (1–3), which directs production of a cell-surface adhesion protein by the retina’s photoreceptor and bipolar cells (4, 5). XLRS is characterized by a moderate to severe decrease in visual acuity (4). Patients with XLRS experience splitting primarily in the inner nuclear layer of the macula, and in the periphery in about 50% of patients (6, 7). This results in abnormal communication between the photoreceptors and bipolar cells, causing a reduced b-wave, and often an electronegative electroretinogram (ERG), defined as a b-wave below the baseline (4, 8, 9). XLRS currently lacks an effective treatment. However, to mitigate macular schisis, various approaches have been employed, including the utilization of topical and oral carbonic anhydrase inhibitors (10). Research conducted on mice lacking *Rs1* function has demonstrated that the use of recombinant adeno-associated virus (rAAV) gene therapy vectors expressing functional *RS1* can lead to substantial improvements in both retinal structure and function (11–13) including photoreceptor preservation, a decrease in the number and size of schisis cavities, and a recovery of the ERG b-wave response (5, 11, 12, 14–17).

Given the promising outcomes observed in preclinical studies using animal models, this approach led to clinical trials involving patients with XLRS. A phase I/IIa clinical trial to assess the safety and tolerability of ocular gene therapy using an AAV8-*RS1* vector, administered intravitreally, was performed with three different doses (1E9, 1E10 and 1E11 vg/eye) (18). Another phase I/II dose-escalation study to assess safety and potential efficacy in patients with XLRS utilized a vector with capsid residue modification, rAAV2-tYF-CB-h*RS1*, intravitreally (1E11, 3E11, and 6E11 vg/eye). Both studies showed that high dose IVT injection causes ocular inflammation (18, 19). The observed ocular inflammation limited substantial improvement in visual function outcomes. The lack of significant improvement underscores the challenges and complexities involved in developing effective treatments for XLRS using gene therapy approaches. These findings emphasize the need for further research and refinement of therapeutic strategies and the relationship between route of administration, dosing, and efficacy to address the underlying mechanisms and specific limitations associated with XLRS.

The transduction efficiency of AAV is influenced by various stages, including vector binding to receptors on the cell surface (20). Studies have demonstrated that phosphorylation of tyrosine residues on the capsid surface of AAV2 by epidermal growth factor receptor protein tyrosine kinase can trigger ubiquitination and subsequent degradation of viral particles (21). To mitigate this degradation process, researchers have employed site-directed mutagenesis, specifically changing tyrosine-to-phenylalanine (Y-F), on some of the seven capsid surface exposed tyrosine residues in the VP3 common region of AAV2. This modification has been reported to protect vector particles from proteasome degradation, resulting in significant improvements in transduction efficiency compared to the WT AAV2 vector. These enhanced transduction capabilities in AAV2tYF vector have been observed both in tissue culture experiments and animal models (22). Subretinal injection of AAV vectors for gene therapy may have advantages over IVT injection in terms of the humoral immune response. Some authors suggest that IVT injections are more prone to inducing the production of neutralizing antibodies compared to subretinal injections. These antibodies have the potential to impede the successful transfer of genes during gene therapy (23, 24). Although IVT administration offers the potential for panretinal transduction without the difficulties of subretinal surgery, especially attractive in eyes with XLRS in which bullous schisis cavities with holes in inner and/or outer retinal layers may be present, there is growing evidence indicating that IVT injections carry a higher risk of humoral immune responses compared to subretinal injections (23). Considering these factors, subretinal injection may be a preferred route of administration for gene therapy targeting the retina.

Therefore, we conducted a dose-response study using subretinal injection of the optimized rAAV2tYF vector, which contains three tyrosine-to-phenylalanine mutations in the capsid to deliver a functional *RS1* gene to the *Rs1-*KO mouse. Long-term effects of retinal rescue after rAAV2tYF-CB-h*RS1* subretinal gene delivery were investigated.

## 2 Materials and methods

### 2.1 Study design

*Rs1-*KO animals between postnatal day (P) 23–(P) 31 received subretinal injections of rAAV2tYF-CB-h*RS1*. In these treated animals, one eye received the subretinal gene therapy treatment, and the contralateral eye served as the untreated control. To evaluate the effect of the subretinal injection procedure on the retina, additional sham injections delivering the buffer diluent alone were performed. Completely untreated *Rs1-*KO mice were also included. Both male (hemizygous mutant) and female (homozygous mutant) *Rs1*-KO mice were used in this study. Outcome measures include western blotting, ERG, OCT, IHC and VGSA. Western blotting was performed on treated mice at 1 MPI to quantify RS1 protein expression. ERG and OCT were performed on treated mice at 1, 2, 3, 5, 7 and 12 MPI, to evaluate retinal function and photoreceptor survival over time. Immunohistochemistry (IHC) was performed at 13 MPI to evaluate photoreceptor survival on the cellular level. Visually guided swim assay (VGSA) was performed at two different time points (4–6 months of age and 9–11 months of age). Number of eyes enrolled in the dose-response study is shown in Table 1.

**Table 1:** Number of eyes enrolled in the dose-response study.

Number of eyes	Dose (vg/eye)	Study eye	Sex	Age (months)
3	8E7	OD	M	1
4	8E7	OS	M, F	1
5	8E8	OD	M, F	1
2	8E8	OS	M, F	1
4	8E9	OD	M, F	1
3	8E9	OS	M, F	1
6	Buffer diluent sham treatment	OD	M	1
9	Untreated	OD	M, F	1
18	Untreated	OS	M, F	1

### 2.2 Animal husbandry and ethics statement

This study followed the guidelines set forth in the National Institutes of Health’s Guide for the Care and Use of Laboratory Animals. All animal handling procedures were performed in strict accordance with the approved Institutional Animal Care and Use Committee (IACUC) protocol #1041421 of the University of Iowa. The *Rs1-*KO mouse model was generously provided by Paul Sieving, M.D., Ph.D. at the National Eye Institute. The *Rs1-*KO mouse model was generated through homologous recombination in 129Sv/Ev mouse embryonic stem cells using a targeting construct, where a 17.5 kb mouse genomic DNA fragment containing exon 1 and most of intron 1 of the mouse *Rs1* gene was replaced with a neomycin resistance (neoR) gene cassette, resulting in the knockout of the *Rs1* gene. The characterization of this mouse model has been described in detail elsewhere (14). Animals were housed according to IACUC recommendations. Animals were generated by crossing *Rs1-*KO males with either *Rs1-*KO or heterozygous females. Methods of euthanasia used were carbon dioxide inhalation followed by cervical dislocation. Humane endpoints were strictly observed, and every effort was made to minimize suffering.

### 2.3 Genotyping

Genotyping of *Rs1-*KO mice was performed using Taq polymerase (M0273S, New England BioLabs) using the primers listed in Table 2 following the manufacturer’s instructions. The PCR products were measured by agarose gel electrophoresis (E-gel Invitrogen by Thermo Fisher Scientific, California, United States). The 10 μL PCR reaction included 2 μL of betaine. The cycling conditions consist of 5 min of initial denaturing step followed by 35 cycles of denaturing step at 94°C (30 s), the annealing step at 57°C (30 s), extension at 72°C (30 s), and then 4 min of final extension at 72°C. The size for the WT band in the PCR product is 516 base pair (bp), and for the knockout band is 300 bp.

**Table 2:** Primers for *Rs1*-KO genotyping.

Primer	Ratio	Sequence (5′ - > 3′)
*Rs1*-KO-WT-Fr	33%	TAGGGGCCCACATCTTCCAAC
PLA2 (13)	33%	GTTCTTCGGACGCCTCGTCAACAC
*Rs1*-KO-WT-Rv	33%	GTGACAAAGAGCCACACAACAGTGACC

### 2.4 AAV packaging and subretinal injection

**Figure 1:**
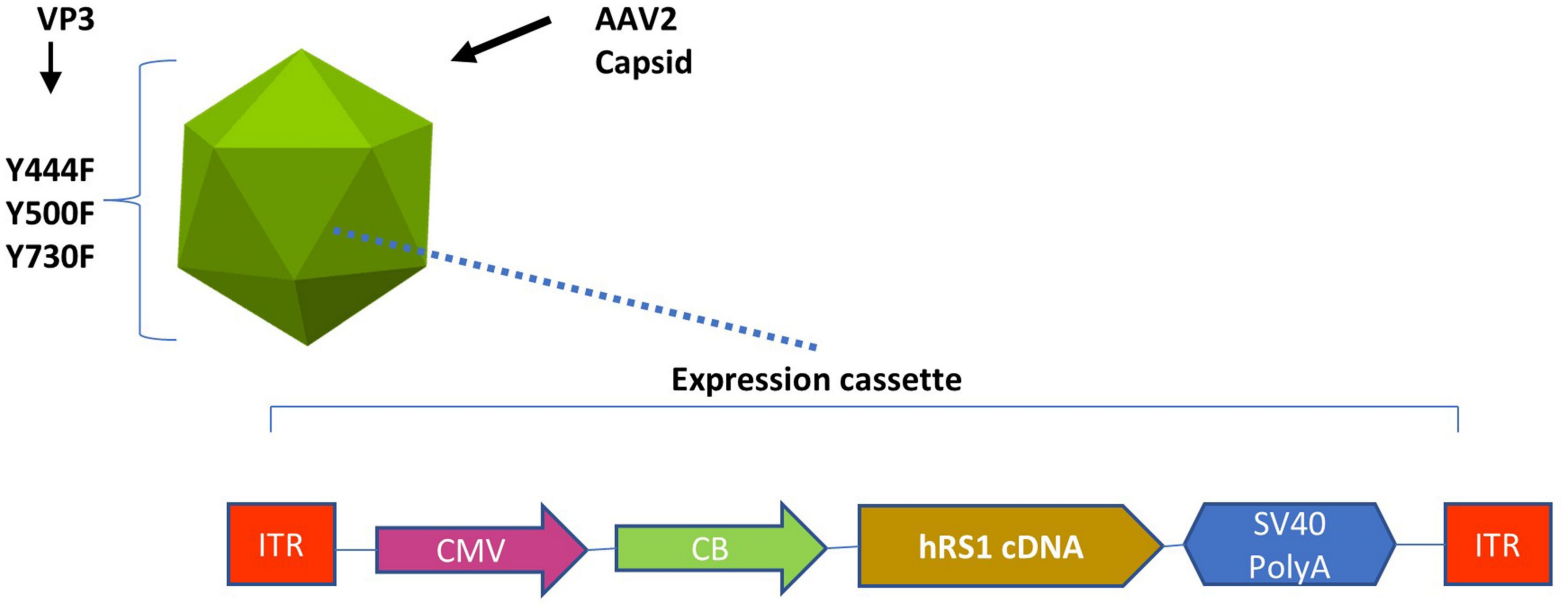
Expression cassette of the rAAV2tYF-CB-h*RS1* vector. Components of rAAV2tYF-CB-h*RS1*, the vector contains AAV serotype 2 inverted terminal repeats (ITRs) and an expression cassette consisting of a cytomegalovirus enhancer (CMV) and chicken β-actin promoter (CB), human *RS1* complementary DNA, and a simian vacuolating virus 40 polyadenylation (SV40 PolyA) sequence and is packaged in an AAV2 capsid containing tyrosine-to-phenylalanine mutations in 3 surface-exposed tyrosine residues (Y444F, Y500F, Y730F) in the VP3 protein capsid (rAAV2tYF).

rAAV2tYF-CB-h*RS1* was manufactured in compliance with current good manufacturing practices by a contract manufacturer under the direction of Applied Genetic Technologies Corporation (19). This vector was a gift from *TeamedOn*, Rockville, MD. The vector contains AAV serotype 2 inverted terminal repeats and an expression cassette consisting of a cytomegalovirus enhancer and chicken β-actin promoter, human *RS1* complementary DNA, and a simian vacuolating virus 40 (SV40) polyadenylation (polyA) sequence and is packaged in an AAV2 capsid containing tyrosine-to-phenylalanine mutations in three surface-exposed tyrosine residues (rAAV2tYF) (22). This expression cassette is shown in Figure 1.

For subretinal injection, Mice were anesthetized by intraperitoneal injection using a mixture of ketamine and xylazine (87.5 mg/kg ketamine, 12.5 mg/kg xylazine) at a volume of 0.1 mL per 20 g body weight with a concentration of 17.5 mg/mL ketamine and 2.5 mg/mL xylazine. Subretinal injections were performed with a 32-gauge Hamilton syringe under a Zeiss OPMI f 170 surgical microscope as described previously (25–27). For rAAV2tYF-CB-h*RS1*, two μL of virus at 4E9 or 4E8 or 4E7 vg/μL concentrations were injected into the temporal subretinal space of the mice eyes. The dilution buffer used was a mixture of Alcon BSS and 0.014% (v/v) Tween 20. Alcon BSS, known as BSS^®^ Sterile Irrigating Solution, is a sterile, balanced salt solution. It contains the following components per milliliter: 0.64% sodium chloride (NaCl), 0.075% potassium chloride (KCl), 0.048% calcium chloride dihydrate (CaCl_2_·2H_2_O), 0.03% magnesium chloride hexahydrate (MgCl_2_·6H_2_O), 0.39% sodium acetate trihydrate (C_2_H_3_NaO_2_·3H_2_O), 0.17% sodium citrate dihydrate (C_6_H_5_Na_3_O_7_·2H_2_O), and pH-adjusting agents like sodium hydroxide and/or hydrochloric acid to maintain a pH of around 7.5. Additionally, it is formulated with water for injection and has an osmolality of approximately 300 mOsm/kg. Subretinal treatment alternated between the right (OD) and left (OS) eyes for different cohorts of animals, and the contralateral eyes served as untreated controls.

### 2.5 Exclusion criteria

The subretinal injection procedure involves temporarily separating the retina from the retinal pigment epithelium (RPE) to create a subretinal bleb. The success of the subretinal injection can be determined by the presence and the quality of the subretinal bleb immediately after the injection. At the time of the subretinal injections, the quality of the retinal blebs were visually assessed by the injector for each animal under the Zeiss OPMI f 170 surgical microscope. All mice with visible blebs were included in the study. Rarely, the subretinal injection causes a large vitreous hemorrhage, or a chronic retinal detachment detectable on OCT at 1 MPI. These eyes were excluded. A specific exclusion criterion was set for the VGSA: in rare cases, some mice displayed a lack of motivation to swim and instead repeatedly floated in the pool during the experiment. To ensure data quality, any data from a mouse (both light and dark testing) were excluded if its mean swim time deviated by more than 1 standard deviation from the group’s median due to consistent floating behavior (floating more than three times per test episode or requiring more than three interventions to stop floating, achieved either by snapping fingers to create an audible sound or by flicking the mouse’s tail).

### 2.6 Western blotting

Mice were euthanized by CO_2_ asphyxiation followed by cervical dislocation. Mouse eyes were enucleated, and the anterior chamber and the lens were removed using micro-dissecting scissors. The retinas of 2 WT eyes, 2 buffer treated eyes, 3 eyes from each of the 3 dose groups and 1 untreated eye were separated from the pigmented retinal epithelium with forceps, snap-frozen in liquid nitrogen, and stored in −80°C. Retinas were lysed using RIPA lysis and extraction buffer (Thermo Fisher Scientific), protease and phosphatase inhibitor mixture (Thermo Fisher Scientific) and gently grinded using homogenizing pestles (Research Products International). Homogenates were centrifuged at 16,000 × *g* for 10 min at 4°C, and supernatants were aliquoted. Protein concentrations were measured with a DC Protein Assay kit (Bio-Rad) following the manufacturer’s instruction. 4× NuPAGE LDS sample buffer (Thermo Fisher Scientific), and 10× reducing agent (Thermo Fisher Scientific), were added to 18 μg of total proteins. Samples were boiled for 5 min, loaded and separated on a 4%–12% (w/v) NuPAGE Bis-Tris gel (Thermo Fisher Scientific) and transferred to a nitrocellulose membrane (Bio-Rad). Proteins were detected by primary antibodies: polyclonal antibody against human RS1 (Atlas Antibodies, HPA059546, dilution 1:750) and β-Actin antibody monoclonal IgG sc-47778 (Santa Cruz Biotechnology, dilution 1:1000) then secondary antibodies: IRDye^®^ 800CW goat anti-rabbit IgG and 680RD goat anti-mouse IgG, dilutions 1:10000 (LI-COR). Images were taken with LI-COR Licor Odyssey CLx 9,140 Imaging System Unit2 Pred DLx—AV and quantified with Image J software.

### 2.7 Electroretinography

Electroretinography (ERG) was obtained using the Celeris system from Diagnosys (Diagnosys LLC, MA, United States) 1 month after treatment initiation and again at 2, 3, 5, 7, and 12 MPI. Before conducting the ERG procedure, the mice underwent overnight dark adaptation. Mice were anesthetized by intraperitoneal injection using a mixture of ketamine/xylazine as described above. The eyes were treated with a 1% tropicamide ophthalmic solution (Akron Inc., Lake Forest, IL) 3 min prior to the ERG test. ERGs were recorded simultaneously from the corneal surface of each eye. Before placing the electrodes, GONAK Hypromellose ophthalmic demulcent solution (NDC 17478-064, Akorn) was applied to the eyes. Body temperature was maintained at a constant temperature of 38°C using the system heat pad. Dim red light was used for illumination until DA testing was completed. To assess rod function, dim flashes of 0.01 cd·s/m^2^ (0.01 dim flash) were delivered under DA conditions. For combined rod-cone function assessment, bright flashes of 3.0 cd·s/m^2^ (3.0 SCR) were administered while the mice remained in a DA state. To evaluate cone function, the mice were LA for 10 min to bleach the rods in the retina. Then, two different measures were used to isolate the functions of cones: 3.0 cd·s/m^2^ single flashes (3.0 Flash), and a flickering light at 5 Hz of 3.0 cd·s/m^2^ intensity (5 Hz flicker). These standardized ERG testing protocols were adapted for mice from the guidelines established by the International Society for Clinical Electrophysiology of Vision (ISCEV) (28).

### 2.8 Optical coherence tomography

Optical coherence tomography (OCT) was performed using the spectral domain (SD) Envisu Image Guided SD-OCT system. Bioptigen InVivoVue software (Leica Biosystems Inc., Buffalo Grove, IL), was used for quantification and allowed segmentation and thickness estimation for all retinal layers (26). Prior to the OCT procedure, mice were anesthetized by intraperitoneal injection using a ketamine/xylazine mixture as described above, and their pupils were dilated with 1% tropicamide ophthalmic drops. The mice were then placed in the animal imaging mount-rodent alignment stage (AIM-RAS) setup for image acquisition (29). Axis manipulation before image capture was done to centrally align the optic nerve head (ONH) in the middle of the scans as a landmark. All images are a central OCT scan from temporal to nasal sides of the mouse. The quantification of the outer nuclear layer (ONL) thicknesses in both control and *Rs1*-KO mice, and the cyst severity score, were measured and averaged from 4 locations 500 μm equidistant from the center of ONH using the in-software calipers provided by Bioptigen. This cyst scoring system is modified based on the one reported by Bush et al. (30). Cyst severity scores were assigned based on the height of schisis cavities in the retinas [(1) no cavities, (2) <30 μm, (3) 30–49 μm, (4) 50–69 μm, (5) 70–99 μm, (6) ≥100 μm] Intraperitoneal antisedan (atipamezole hydrochloride 5.0 mg/mL) 0.2 mL per 20 g body weight injection was given for reversal of anesthesia.

### 2.9 Immunohistochemistry

Immunostaining and imaging techniques, as previously detailed (31), were employed for eye sections. Initially, sections underwent permeabilization with 0.3% Triton X-100 in PBS for 10 min at room temperature. Subsequently, blocking occurred with a buffer consisting of 5% BSA, 5% normal goat serum, and 0.05% Triton X-100 in PBS. The sections were then incubated with the biotinylated-peanut agglutinin (biotinylated-PNA) (Vector Laboratories #B-1075; 1:500 dilution), in a dilution buffer containing 5% BSA, 1% normal goat serum, and 0.05% Triton X-100 in PBS at 4°C overnight. Following washing, secondary antibodies or streptavidin Alexa Fluor-568 conjugate were applied at room temperature for 1 h. After additional washing, sections were mounted using Vectashield mounting medium with DAPI (Vector Laboratories), and images were captured using an Olympus IX71 microscope. Six total images were obtained per eye, with three serial images on either side of the optic nerve at 40× magnification. Three individuals, who were blinded to treatment groups quantified the images independently. Quantifications, averaged and divided by the image width, yielded reported average cones per 100 μm. Whole eye images were captured at 4× magnification.

### 2.10 Visually guided swim assay

The visually guided swim assay, described in detail elsewhere (32), was modified from the Morris water maze (33) and incorporated features of the mouse swim assay developed by Pang et al. (34). Briefly, mice were placed in a plastic pool full of water containing a randomly placed platform. They were trained to know there would be a platform in the pool they could climb upon. The experiment was conducted under two different lighting conditions: normal room light (13.35 cd/m^2^) and dark with dim red lighting (approximately 4.17 × 10^−3^ cd/m^2^). The protocol involved 4 training days in the light, followed by 4 testing days in the light, then 2 training days in the dark, and finally 4 testing days in the dark. Each day, mice performed five swim trials, and the platform locations were the same for all mice in the group. A time limit of 60 s was set for each trial to prevent fatigue. If a mouse failed to reach the platform within 60 s, its time was recorded as 60 s for that particular trial.

### 2.11 Statistical analysis

Statistical analysis was conducted using GraphPad PRISM 10.1 (GraphPad Software, Inc., San Diego, California, United States). Two-way ANOVA was employed to examine group differences over time for ERG quantification, OCT scan measurements and VGSA, followed by the non-parametric multiple comparisons *post hoc* Tukey’s test. Student’s *t*-test was utilized to compare control and *Rs1*-KO mice. Two-way ANOVA test followed by Dunnett’s multiple comparison *post hoc* test was used to analyze the difference between the 8E8 vg/eye dose treated and WT or heterozygous control groups. One way ANOVA was employed for the IHC quantifications, followed by the non-parametric multiple comparisons *post hoc* Tukey’s test. Values shown are averages ± standard deviation.

## 3 Results

### 3.1 Phenotypic characterization of *Rs1*-KO mouse model reveals key features of XLRS pathology

The *Rs1*-KO mouse model has been shown as an invaluable tool for studying the involvement of the *RS1* gene in a variety of physiological retina processes (4, 5, 11–13). Confirming and adding to previously reported phenotypic descriptions of the natural history and characteristics of the *Rs1*-KO mouse model is crucial for elucidating the underlying mechanisms of XLRS and developing potential therapeutic interventions.

**Figure 2:**
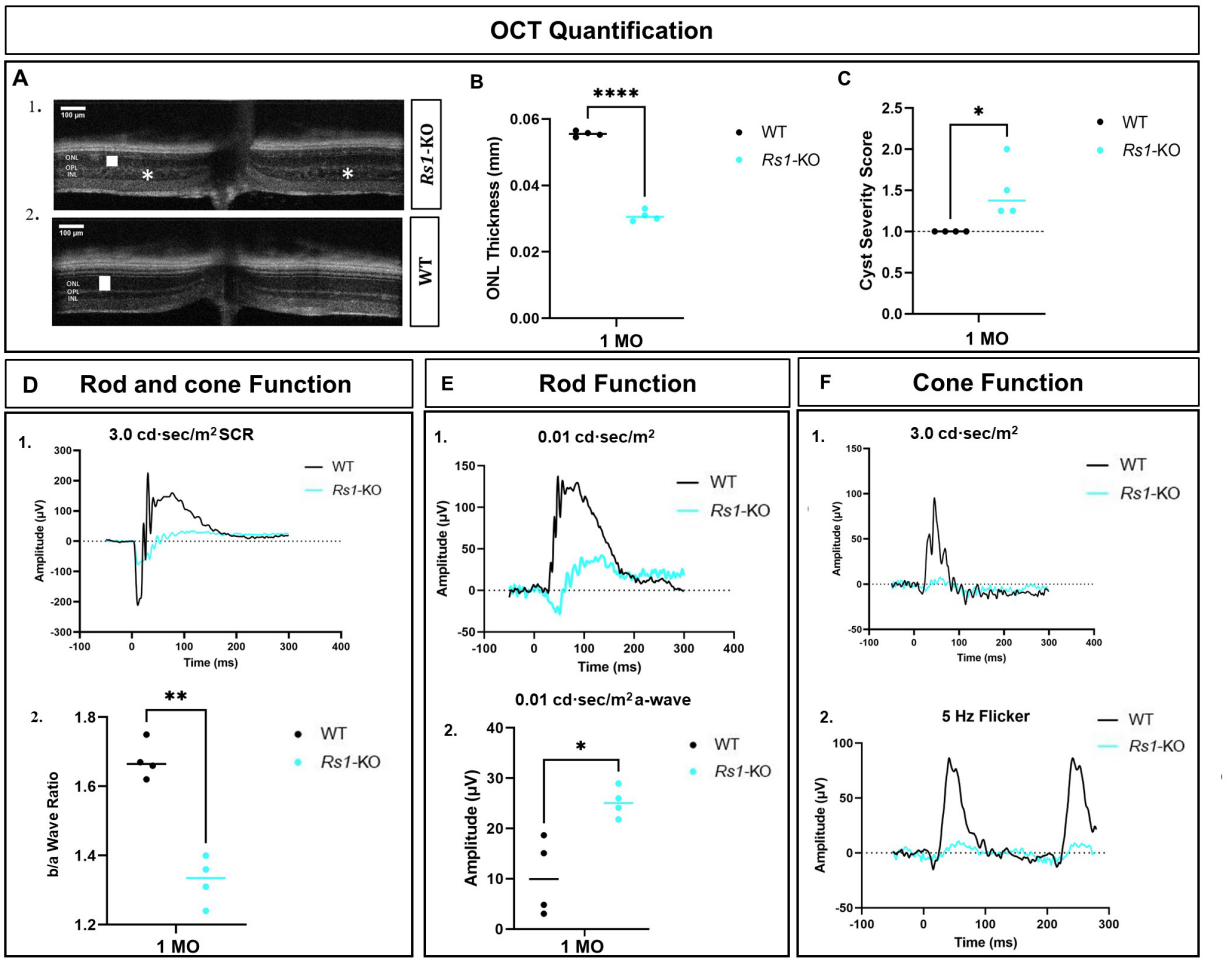
Characterization of retinal phenotypes in retinoschisin-1 knockout mice: rod and cone photoreceptors functional loss and cyst formation. **(A)** OCT images of a *Rs1-*KO mouse (1) and a wild-type (WT) control littermate (2) are shown at 1 month of age. The outer nuclear layer (ONL) thicknesses are indicated by a white solid bar, and cysts are indicated by white stars on the OCT image. **(B)** Quantification of ONL thicknesses in *Rs1*-KO and WT or heterozygous control mice at 1 month of age. *Rs1*-KO mice have thinner ONLs. **(C)** Quantification of the cyst severity in *Rs1*-KO and WT or heterozygous control mice. Cyst severity score is modified from Bush et al. (30) where (1) no cavities; (2) <30 μm; (3) 30–49 μm; (4) 50–69 μm; (5) 70–99 μm; (6) ≥100 μm. *Rs1*-KO mice have cysts whereas WT or heterozygous do not. **(D)** (1) Waveforms in dark-adapted (DA) standard combined response (SCR) ERG using 3.0 cd·s/m^2^ stimuli in *Rs1*-KO and WT or heterozygous control mice. (2) b/a wave ratio is reduced in *Rs1*-KO compared to WT or heterozygous controls. **(E)** At 1 month of age, *Rs1*-KO mice have greater a-wave amplitudes compared to WT or heterozygous control mice after 0.01 cd·s/m^2^ stimuli in DA conditions. Representative waveforms are shown in (1), and the statistical comparison of their a-wave amplitudes is shown in (2). **(F)** Cone function on ERG measured after light adaptation using 3.0 flash (1) and 5 Hz flicker (2). Amplitudes are reduced in *Rs1*-KO compared to WT or heterozygous control mice. MO: month old, *Rs1*-KO: retinoschisin-1 knockout, WT: wild-type or heterozygous mice, μV: Microvolt, *p < 0.05, **p < 0.01, and ****p < 0.0001. Scale bar, 100 μm.

At the age of 1 month, the ONL thickness in *Rs1-*KO mice was 48% (Figures 2A1,B) as thick as the WT or heterozygous controls (Figures 2A2,B) suggesting that photoreceptor degeneration occurs at an early age in the *Rs1-*KO mouse model. In addition to the reduced thickness of the ONL, *Rs1*-KO mice at 1 month of age exhibited schisis (cysts) in the inner nuclear layer compared to the absence of schisis in the WT littermates (Figures 2A,C), (*p* < 0.05).

At 1 month of age, there is a reduction in the amplitudes of the b-wave of the 3.0 SCR ERG as shown by the representative waveform (Figure 2D1). Notably, b/a ratio, the magnitude of the b-wave compared to the magnitude of the a-wave, is reduced (Figure 2D2), similar to ERG findings reported in human XLRS (26). An interesting phenomenon in the untreated *Rs1*-KO eyes is the hyper-normal a-wave amplitudes in the 0.01 dim flash ERG protocol. This was surprising given the fact that these eyes have only about half the number of photoreceptor cells compared to unaffected controls, and the a-wave is generated primarily by photoreceptor cells (35). These amplitudes were much larger than those observed in normal WT or heterozygous mice under the same test conditions with a statistical difference (*p* = 0.0113) (Figures 2E1,2) The hyper-normal a-wave response, possibly due to disrupted synapses augmenting photoreceptor signals for enhanced retinal response, is a feature of the XLRS mouse model that has not been previously reported. Representative waveforms (Figure 2F) show a notable reduction in cone-dependent ERGs in 3.0 flash and the 5 Hz flicker ERG. These findings highlight the involvement of *RS1* in maintaining the functionality of both rod and cone photoreceptors and their ability to generate robust electrical responses. Additionally, these findings show that a reduction in the thickness of the ONL, the appearance of schisis, and a decrease in b/a ratio in ERGs are already present in *Rs1-*KO mice prior to the administration of gene therapy.

### 3.2 Human RS1 protein expression in the retinas of *Rs1*-KO mice is reconstituted in a dose-dependent manner

**Figure 3:**
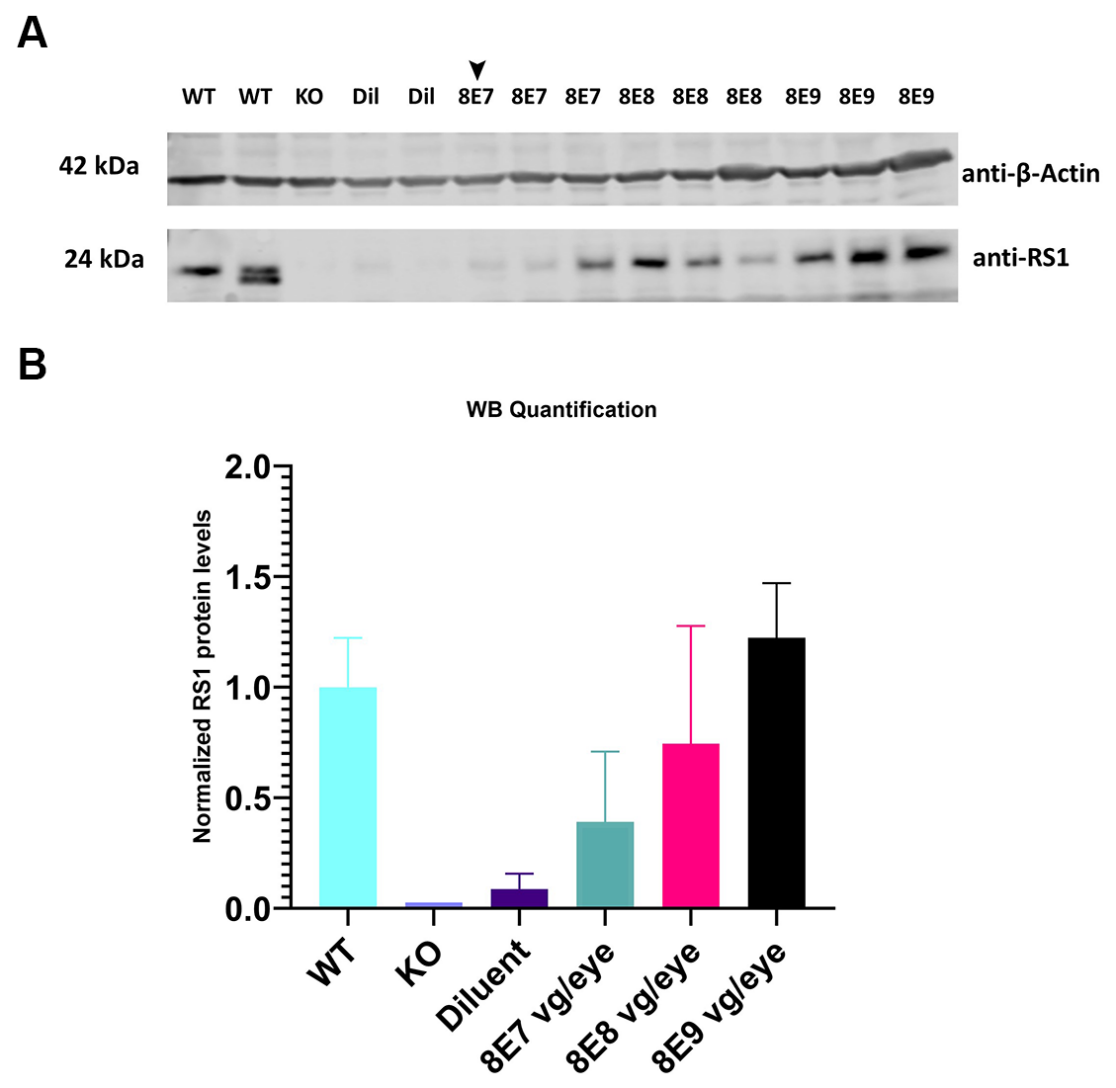
Expression of human RS1 protein in treated retinas of *Rs1*-KO mice reveals a dose-dependent response. **(A)** Immunoblot analysis of eyes treated with 8E9 vg, 8E8 vg, 8E7 vg doses of the AAV2tYF vector, and diluent-treated samples (Dil) retinal protein extracts. Bleb qualities were excellent for all treatment groups except for one retina treated with 8E7 dose that had a very good bleb (arrowhead). Completely untreated *Rs1*-KO and WT retinas served as negative and positive controls. Each lane represents an individual eye, with three eyes analyzed per dose, 2 eyes for sham treatment, 2 eyes for WT, and 1 eye for untreated KO. Eighteen μg of protein were loaded per lane. The expected molecular weights for proteins are indicated on the left, and the antibodies used to visualize the proteins are indicated on the right. **(B)** RS1 expression levels relative to completely WT eyes. Quantification involved normalizing the RS1 band intensity to the actin band intensity for each sample, with the expression level presented as a percentage of WT retinal samples which is set to 100%. The results illustrate a dose-dependent increase in RS1 protein expression in *Rs1*-KO mouse retinas treated with 8E7, 8E8, and 8E9 vg of rAAV2-tYF-CB-h*RS1*.

Different doses of rAAV2-tYF-CB-h*RS1* viral vector (Figure 1) were subretinally delivered to *Rs1*-KO mice between ages of P23–P31. Eyes that received 8E9, 8E8, 8E7 vg/eye of rAAV2-tYF-CB-h*RS1* viral vector were compared to eyes that received buffer diluent only (sham-treated), as well as completely uninjected eyes. A study from this laboratory has previously demonstrated the beneficial effects of buffer injection alone on *RS1* phenotypes (31), necessitating the inclusion of a control group consisting of eyes subretinally injected with buffer/diluent, in addition to uninjected eyes. To demonstrate the expression levels of RS1 protein after administration of different doses of the gene therapy vector, retinas were lysed, and western blotting was performed at 1 MPI (Figure 3A). Each sample consisted of 1 retina. As positive and negative controls, completely WT retinas and *Rs1*-KO retinas were also included. The band intensity of RS1 was normalized to the band intensity of actin for that sample, and then the expression level of each sample was expressed as a percent of the expression level of the WT retinal samples. The results show a dose-dependent increase of RS1 protein expression in the retinas of *Rs1-*KO mice receiving 8E7, 8E8, and 8E9 vg of rAAV2-tYF-CB-h*RS1*. The 8E9 vg dose group had 1.6-fold higher expression than 8E8 vg dosage group, and 8E8 vg dosage group had 1.8-fold higher than 8E7 vg dosage group. Compared to the expression level of RS1 in WT retinas, which is set to 100%, The quantification of western blot data revealed that the RS1 protein expression level after injection of the 8E8 dose is 74.4% of the WT. In contrast, the RS1 protein expression after injection of the 8E9 dose is 122.3% of WT, while the expression in the 8E7 dose injected group is 39% of RS1 WT protein expression (Figure 3B). These findings indicate that the subretinal injection of the rAAV2-tYF-CB-h*RS1* enabled the expression of RS1 proteins in a dose-dependent manner.

### 3.3 Subretinal gene therapy of *Rs1*-KO with 8E8 vg/eye dose rescues cone electrical function

**Figure 4:**
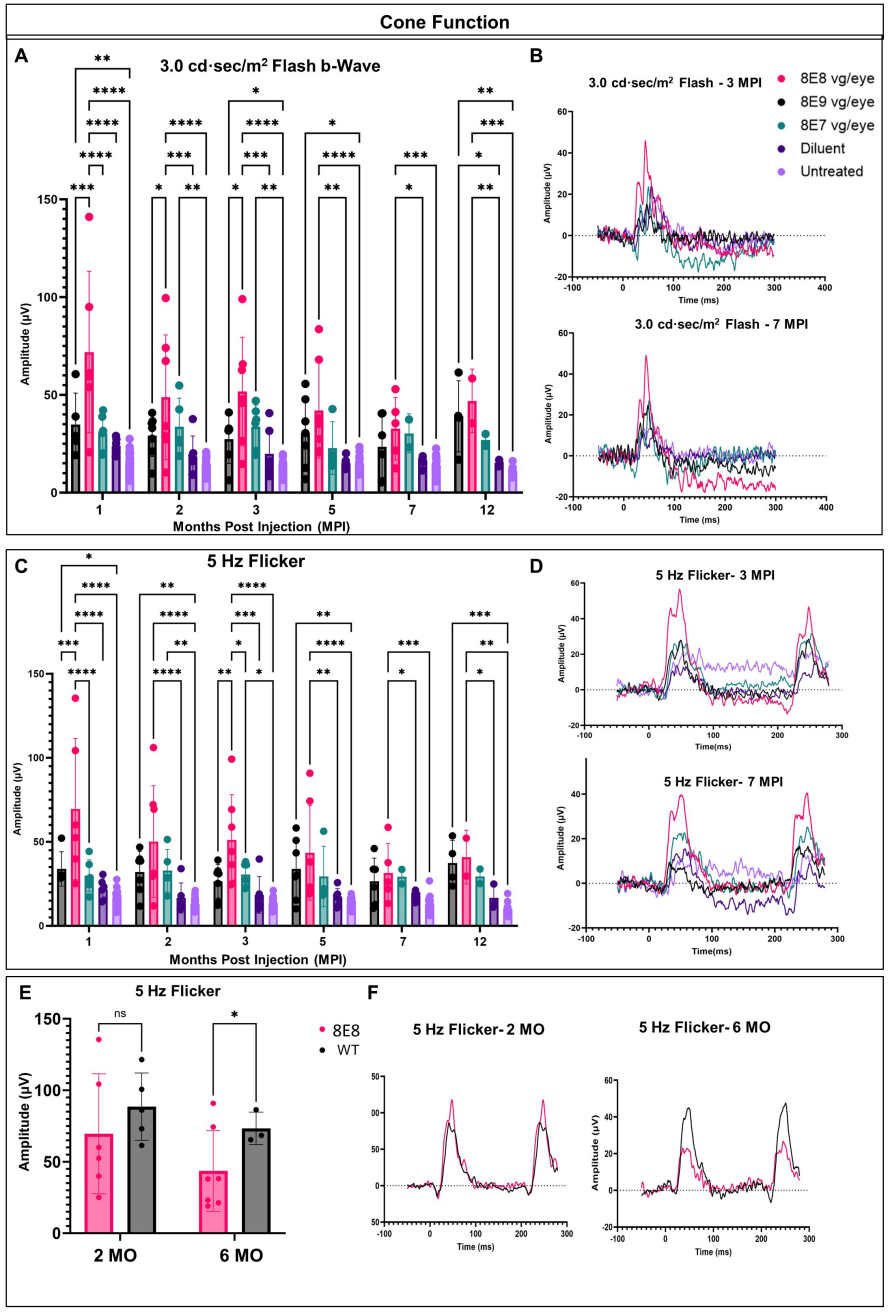
rAAV2tYF subretinal gene therapy improves and sustains cone photoreceptor function in *Rs1*-KO mice treated with the medium dose of 8E8 vg/eye over the course of 12 months. **(A)** Cone-dependent retinal function, assessed through light-adapted (LA) electroretinography after subjecting the eyes to a 3.0 cd·s/m^2^ bright flash. Eyes treated with the 8E8 vg dose consistently displayed significantly greater b-wave amplitudes compared to sham-treated eyes and untreated eyes over 1 year. Eyes treated with 8E9 showed significance compared to the sham-treated eyes only at the experimental endpoint. **(B)** Representative LA waveforms were shown for eyes treated with different doses, as well as for sham-treated, and untreated eyes at 3 MPI and 7 MPI, respectively. **(C)** Another metric used to evaluate cone-dependent retinal function was the 5 Hz flicker test, which consistently showed that 8E8 vg dose-treated eyes outperformed sham-treated and untreated eyes throughout the treatment duration. Neither 8E9 nor 8E7 vg doses showed significance compared to the sham-treated eyes over the treatment course. **(D)** When subjected to a 5 Hz flicker, cones in treated eyes elicited higher amplitudes at 3 MPI, which was not observed in the sham-treated eyes or the untreated contralateral eyes. Over the course of 7 months, treated eyes retained significantly better amplitudes in cone ERGs. **(E)** Comparison of ERG flicker amplitudes between mice that received the medium 8E8 vg dose and WT or heterozygous control mice. At 1 MPI (2 MO), treatment with the 8E8 vg dose restored ERGs to near-WT amplitudes. **(F)** Waveforms from an eye that received the medium 8E8 dose and a WT eye. MO, months old, WT: wild-type or heterozygous mice, µV: Microvolt, *p < 0.05, **p < 0.01, ***p < 0.005, and ****p < 0.0001.

In each animal, one eye was treated, and the contralateral eye was left untreated as an internal comparison. We previously showed in Figure 2 that the ERGs in *Rs1*-KO mice are at very low amplitudes at 1 month of age. To evaluate the long-term impact of subretinal gene therapy on retinal function, we conducted ERG tests at 1, 2, 3, 5, 7 and 12 MPI. These assessments measured the function of cones and rods in treated and untreated *Rs1*-KO eyes. Cones are pivotal for daytime vision, color perception, and high-detail sight in humans. We employed LA 3.0 cd·s/m^2^ single-flash and 3.0 cd·s/m^2^ 5 Hz flicker ERG to evaluate cone function in both groups. At 1 MPI, treated *Rs1-*KO eyes with the dose 8E8 vg/eye had a strong response to the 3.0 flash and had higher b-wave values (71.86 ± 41.29 μV, *n* = 7) compared to both sham-treated (23.85 ± 3.98 μV, *n* = 6, *p* < 0.0001) and untreated eyes (16.43 ± 5.13 μV, *n* = 23, *p* < 0.0001). This improvement was also observed at 2, 3, 5 and 7MPI (Figure 4A). The group that was treated with the dose 8E9 vg showed higher b-wave values compared to the untreated eyes at 1, 3 and 5 MPI (*p* = 0.006, *p* = 0.04 and *p* = 0.03 respectively) and the group that was treated with the 8E7 vg dose had higher b-wave values compared to the untreated eyes at 2 and 3 MPI (*p* < 0.05). However, neither of the groups treated with either doses result in any significant difference in amplitudes compared to the sham-treated eyes in response to the LA 3.0 flash test (Figure 4A). It is worth noting that the group treated with the medium dose (8E8 vg/eye) showed statistical significance in response to the 3.0 flash test compared to the other groups treated with 8E9 vg and 8E7 vg doses at 1 MPI, *p* < 0.0001 and continued to show significance compared to 8E9 dosage group at 2 and 3 MPI (*p* < 0.05) (Figure 4A). This data shows that the dose 8E8 of rAAV2tYF gene therapy restores the electrical function in cones and is superior to the other doses.

Subsequently, we investigated whether the restoration of cone functions persisted in the 8E8 treated eyes. At the endpoint of the study (12 MPI), treated eyes had higher b-wave amplitudes in response to 3.0 flash test compared to untreated eyes (Figure 4A; 8E8 treated eyes: 46.94 ± 16.2 μV, *n* = 2; untreated eyes: 11.11 ± 2.3 μV, *n* = 11; *p* = 0. 0001) and compared to the sham-injected eyes (Figure 4A; sham-treated eyes: 15.1 ± 1.6 μV, *n* = 3; *p* = 0. 005). Amplitudes of eyes that received the 8E7 vg dose were not different than amplitudes of those that received the buffer/diluent or those of uninjected eyes. On the other hand, eyes treated with the 8E9 vg dose also had significantly higher amplitudes (39.01 ± 18.21 μV, *n* = 4) compared to sham-injected eyes (*p* = 0.04) and uninjected eyes (*p* = 0.001) (Figure 4A). However, those that received the 8E8 vg dose had a higher response on average, speaking to the potential importance of gene dose in gene therapy development for *RS1*. The light adapted 3.0 flash stimulus light elicited a high waveform in treated eyes, whereas untreated contralateral eyes and sham-injected eyes displayed poor responses (Figure 4B, 3 MPI and 7 MPI), suggesting that the regained cone electrical function was partially retained in vector gene therapy treated eyes over the course of the experiment. The waveforms of the 8E8 vg treated eyes showed superiority over the other groups treated with other gene therapy doses (8E9 vg and 8E7 vg) (Figure 4B).

The LA 5 Hz flicker test, another metric, was conducted for measuring cone electrical function in mice. This test exclusively measures cone function since the frequency of the flicker is more rapid than the recovery time of rods. At 1 MPI, the medium dose (8E8 vg) treated group showed a significant improvement in the amplitudes of the 5 Hz flicker ERG compared to the sham-treated eyes (*p* < 0.0001) and the untreated eyes (*p* < 0.0001). Eyes that received the 8E9 vg dose had higher amplitudes compared to uninjected eyes, but not compared to sham-injected eyes. Comparison with sham-injected eyes is important for determining the efficacy of gene therapy, since unexpected beneficial effects were observed after injection of buffer/diluent alone in *Rs1-*KO mice (31). The improvement in cone function by the 5 Hz flicker test was not limited to the 1 MPI time point but was consistently observed at 2, 3, 5 and 7 MPI (Figure 4C). Treated eyes with 8E8 vg dose demonstrated a clear periodic waveform in response to the 5 Hz flicker (Figure 4D). In contrast, the 5 Hz flicker stimuli had a very poor response in the sham-treated eyes and the untreated eyes at any age (Figure 4D). Therefore, gene delivery was able to reestablish the electrical function in the cone photoreceptor cells in the shorter term (3 MPI). At the experimental endpoint (12 MPI), 8E8 vg dose treated eyes had greater responses to the 5 Hz flicker stimuli compared to untreated eyes (8E8: 40.93 ± 15.9 μV, *n* = 2; untreated eyes: 10.18 ± 3.9 μV, *n* = 11; *p* = 0.001 Figure 3C), and compared to the sham-injected eyes (Figure 3C; 8E8 treated eyes: 31.54 ± 17.49 μV, *n* = 2; sham-treated eyes: 16.65 ± 7.3 μV, *n* = 3; *p* = 0.04). 8E9 vg dose treated eyes also showed statistical significance compared to the untreated eyes at the experimental endpoint (*p* = 0.0009) but not compared to the sham-injected eyes (*p* = 0.08). At 7 MPI, treated mice with the 8E8 vg dose had a robust periodic waveform in their treated eyes, while very small waveforms were observed in the untreated contralateral eyes and the sham-injected eyes (Figure 4D). Therefore, gene augmentation with the dose of 8E8 vg enabled cone photoreceptor cells to restore their electrical function on ERG. Remarkably, when comparing the medium 8E8 vg dosage group to WT or heterozygous control mice at 2 months old (MO) (1 MPI) there was no significant differences between WT eyes and 8E8 vg dose treated eyes (2 MO, *p* = 0.1305; Figure 4E). Though 8E8 vg dose treated eyes were significantly lower than WT at 6 MO (5 MPI) (6 MO, *p* = 0.02; Figure 4E) they remained significantly higher than sham-treated eyes and untreated eyes (Figure 4C) and maintained a robust periodic waveform (Figure 4F). This underscores the substantial impact of the medium 8E8 vg gene therapy dose in restoring cone function to the level seen in WT controls at least at 2 MO.

In response to the 5 Hz flicker test, the high dose treated group (8E9 vg) showed better amplitudes compared to the untreated eyes at 1, 2, 5 and 12 MPI, *p* < 0.05. Also, the low dose treated group (8E7 vg) showed better amplitudes compared to the untreated eyes only at 2 and 3 MPI, *p* < 0.05. However, neither of the treated groups showed any significance compared to the sham-treated eyes (Figure 4C). It is worth mentioning that eyes treated with the 8E8 vg dose showed better amplitudes in response to the 5 Hz flicker test compared to eyes treated with 8E9 vg and 8E7 vg doses at 1 and 3 MPI, *p* < 0.05 (Figure 4C). Importantly, the group treated with isotonic sham injections demonstrated slightly better amplitudes in response to the 5 Hz flicker test compared to untreated eyes, aligning with findings from a prior study by our laboratory (31). However, while the sham-treated eyes performed better than untreated eyes, the observed improvements were of a much lower magnitude than those observed with gene therapy. This underscores the importance of gene augmentation in achieving consistent improvements and the importance of the administered dose.

### 3.4 Subretinal gene therapy with the dose of 8E8 vg/eye slows the loss of rod and cone photoreceptor functions in treated eyes

**Figure 5:**
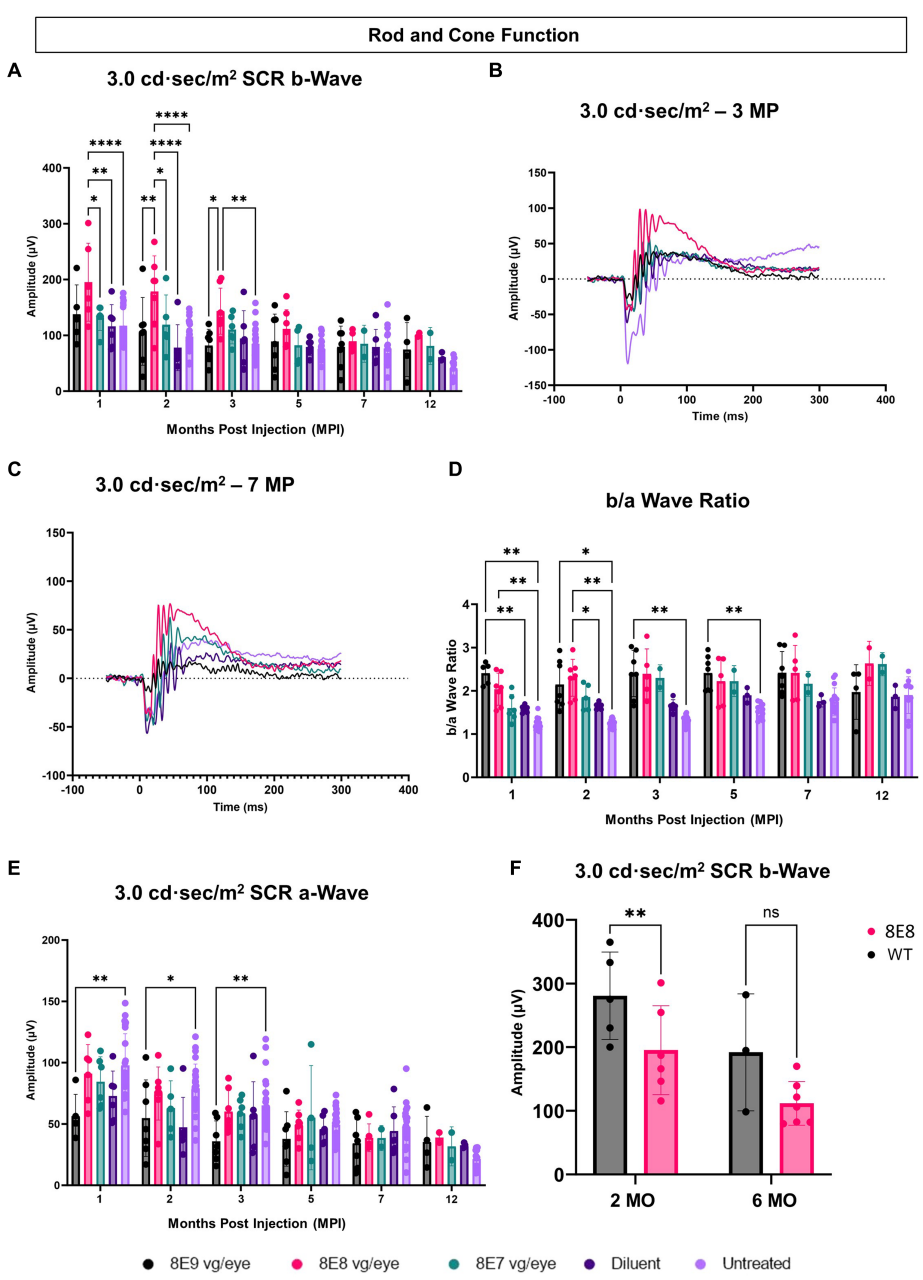
Subretinal gene therapy of *Rs1*-KO delays loss of retinal function in standard combined response ERG. **(A)** Combined rod-cone function was measured by 3.0 cd·s/m^2^ bright flash under dark-adapted conditions, at 1, 2, 3, 5, 7 and 12 MPI. The 8E8 vg dose treated eyes had higher b-wave amplitudes compared to diluent-injected eyes and untreated contralateral eyes. **(B,C)** Representative waveforms of the 3.0 cd·s/m^2^ bright flash SCR ERG test are shown for all vector doses, diluent-injected eyes, and untreated eyes at 3 MPI and 7 MPI, respectively. The 8E8 dose had higher amplitudes at 3 MPI and at 7MPI. Waveforms from the same mice were used at each time point. **(D)** An elevated b/a ratio was observed in the 8E8 vg dose-treated eyes until 2 MPI time point and in 8E9 vg dose treated eyes until 5 MPI. **(E)** SCR a-wave amplitudes of eyes treated with different doses of rAAV2tYF gene therapy vector, sham-treated and untreated control mice. **(F)** Rod and cone function comparison between the medium 8E8 vg dose and WT or heterozygous control mice at 2 and 6 MO. MO, months old, WT: wild-type or heterozygous mice, µV: Microvolt, *p < 0.05, **p < 0.01, and ****p < 0.0001.

We measured the combined function of rod and cone photoreceptors using the 3.0 SCR ERG after exposing DA eyes to 3.0 cd·s/m^2^ bright flashes. The b-wave amplitudes, representing bipolar cell responses driven by photoreceptor input, were significantly higher in the 8E8 vg dose-treated eyes compared to untreated eyes at 1 and 2 MPI (*p* < 0.0001) and 3 MPI (*p* = 0.0016) and compared to the sham-treated eyes at 1 MPI (*p* = 0.0023) and 2 MPI (*p* < 0.0001) (Figures 5A,B). The 8E8 vg dose treated group also showed significantly higher amplitudes compared to the 8E9 vg dose treated group at 2 and 3 MPI (*p* = 0.002 and *p* = 0.011) respectively and compared to the 8E7 dose treated group at 1 and 2 MPI (*p* < 0.05) (Figure 5A). Groups treated with highest, and the lowest doses did not show any significant b-wave improvements in response to the SCR ERG test at any timepoint compared to both sham-treated and untreated eyes (Figure 5A). This suggests that the treatment dose 8E8 vg delayed the loss of rod and cone photoreceptor function and improved bipolar cell activity. While the 8E8 vg dose treated group showed a decreased treatment effect at 7 MPI compared to earlier time points, it still exhibited a superior waveform compared to untreated eyes, sham-treated eyes, and the 8E7 vg and 8E9 vg doses treated eyes (Figure 5C).

The SCR a-wave reflects primarily the combined rod and cone photoreceptor light response (36, 37) and the b-wave reflects postsynaptic bipolar cell activity, albeit driven by photoreceptor input (38, 39). The b/a ratio is known as a useful marker in XLRS to indicate effective signaling between photoreceptors and bipolar cells (40, 41). In RS1, there is a decrease in the b/a ratio. The 8E8 vg dose-treated eyes at 1 and 2 MPI demonstrated an increase in the b/a ratio in the treated eyes compared to the sham-treated eyes and the untreated eyes (Figure 5D). This increase can be attributed to the increase of the b-wave amplitudes and not a decrease in the a-wave amplitudes. Eyes receiving the 8E8 vg dose had a slight reduction in the a-wave amplitude compared to untreated eyes at 1 MPI, but this was not significantly lower than sham-treated eyes (*p* = 0.50) nor untreated eyes (*p* = 0.95) (Figure 5E). In comparison, the b-wave amplitudes of the 8E8 vg treated eyes were significantly greater than sham-treated and untreated eyes; the same was observed at most time points. This observation suggests that signal transmission from the photoreceptors to the bipolar cells was enhanced in eyes treated with the 8E8 vg dose. On the other hand, we also observed an elevation in the b/a ratio within the 8E9 vg dose treated group at 1, 2, 3, and 5 MPI compared to the untreated eyes and at 1 MPI compared to the sham-treated eyes. However, this was not caused by a significant increase in b-wave amplitudes, but rather a decrease in a-wave amplitudes. Eyes that received 8E9 vg exhibited significantly reduced a-wave amplitudes in the SCR ERG at 1, 2 and 3 MPI compared to the untreated eyes, whereas a significant difference was not observed after the injection of other doses (Figure 5E). As previously mentioned, the 8E9 vg treated group did not exhibit any improvement in b-wave amplitudes over sham-injected or untreated eyes (Figure 5A). Thus, this increase in the b/a ratio is attributed to the significant reductions in a-wave amplitudes, and not an elevation of the b-wave amplitudes.

When the 8E8 dose-treated eyes was compared to the WT or heterozygous control group (WT), WT had significantly higher b-wave amplitudes in response to the SCR test at 2 MO but surprisingly not at 6 MO (Figure 5F).

### 3.5 Subretinal gene therapy of *Rs1*-KO with a dose of 8E8 vg/eye preserves rod photoreceptor function short term

**Figure 6:**
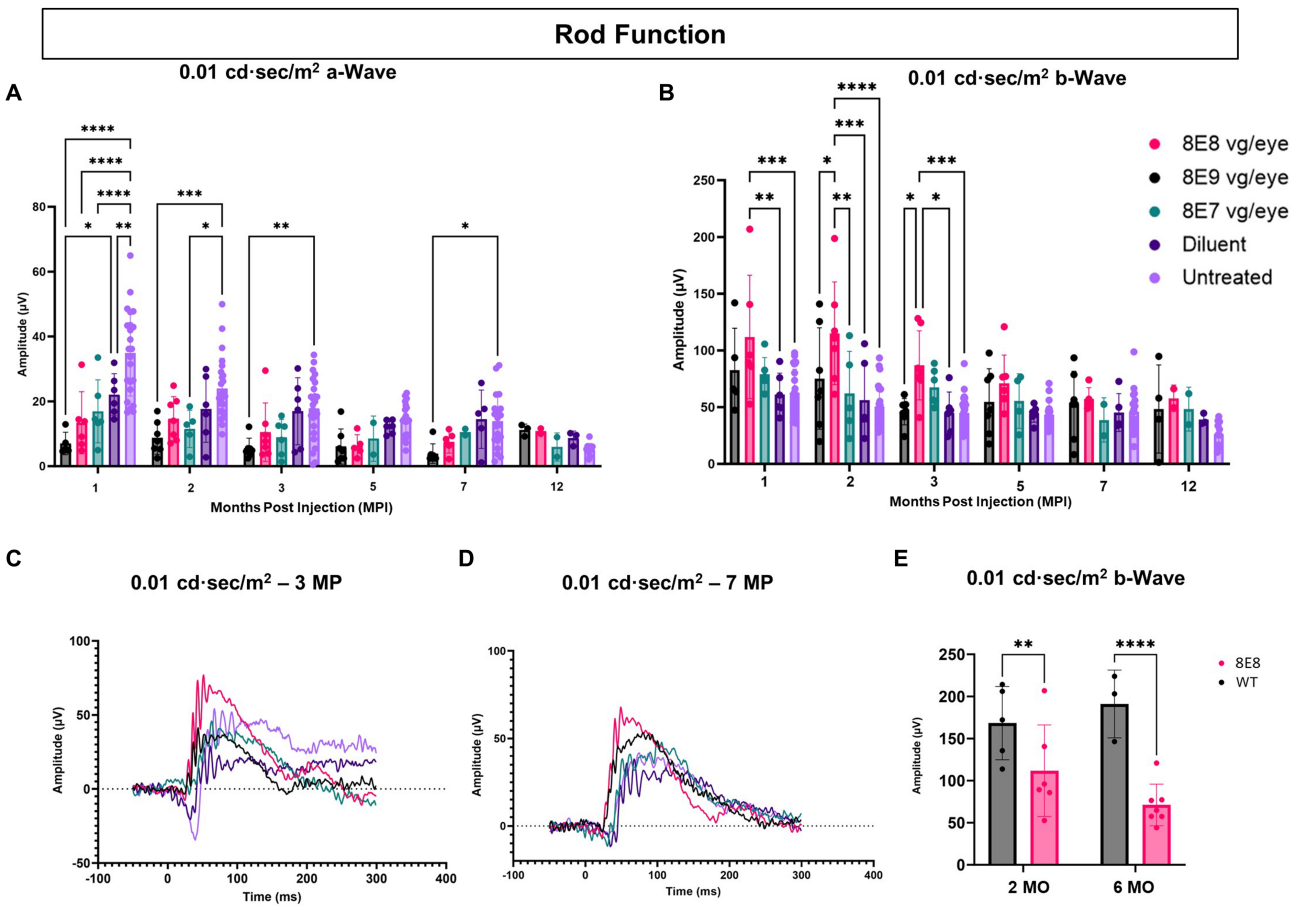
Subretinal gene therapy of *Rs1*-KO with a dose of 8E8 vg/eye delays loss of rod photoreceptor function. **(A)** Rod function was measured by 0.01 cd·s/m^2^ dim flash after DA. Notably, at 1, 2, and 3 MPI, some vector-treated eyes demonstrated a normalization of the hyper-normal a-wave seen in the *Rs1*-KO mouse model (as shown in Figure 2). **(B)** B-wave amplitudes of the 0.01 cd·s/m^2^ dim flash after DA showing the significantly increased b-wave amplitudes of the 8E8 vg dose compared to the sham-treated and untreated eyes. **(C,D)** Waveform comparisons of the 0.01 cd·s/m^2^ dim flash test were made between all vector doses, diluent-injected eyes, and untreated eyes at 3 MPI and 7 MPI, respectively. **(E)** Rod function comparison between the medium 8E8 vg dose and WT or heterozygous control mice (WT) at 2 and 6 MO. MO: months old, WT: wild-type or heterozygous mice, µV: Microvolt, *p < 0.05, **p ≤ 0.005, ***p ≤ 0.0005, and ****p < 0.0001.

Rods are primarily responsible for vision in low-light environments. Rod function was evaluated by subjecting the eyes to a 0.01 cd·s/m^2^ dim flash after overnight dark adaptation. In this manuscript, a novel feature of the *Rs1-*KO mouse—hyper-normal a-wave amplitudes—was reported. At 1 MPI, all treated eyes with all doses had significantly normalized a-wave amplitudes compared to the untreated eyes (*p* < 0.0001) and the 8E9 dose treated group compared to sham-treated eyes (*p* = 0.02) (Figure 6A), suggesting that the hyper-normal a-wave was due to increased electrical activity of photoreceptors in *Rs1*-KO eyes, and not due to differences in the number of rod photoreceptors. Of note, a-wave amplitudes were also partially normalized in diluent-treated eyes at 1 MPI compared to the untreated eyes (*p* = 0.005) (Figure 6A). This observation persisted through most time points except for the sham treated (Figures 6A,C,D). This indicates that the gene therapy using the rAAV2tYF vector successfully corrected the hyper-normal a-wave amplitudes and brought them back to normal levels at most time points. These findings highlight the effectiveness of the rAAV2tYF vector in normalizing a-wave amplitudes in XLRS in the 0.01 dim flash ERG test. Treated *Rs1-*KO eyes receiving the 8E8 vg dose showed a significant improvement in ERG b- wave amplitudes in the 0.01 dim flash ERG test at 1 MPI (111.83 ± 54.36 μV, SD, *n* = 7) compared to sham injections (62.19 ± 18.75 μV, SD, *n* = 6; *p* = 0.0197) and untreated eyes (62.91 ± 20.58 μV, SD, *n* = 23; *p* < 0.0018) (Figure 6B). These improvements were also observed at 2 and 3 MPI (Figures 6B,C) while the 8E9 vg and 8E7 vg doses-treated groups did not show any significant improvements at any timepoint compared to sham-treated or untreated eyes. The 8E8 vg dose-treated group had higher b-wave amplitudes compared to the 8E9 vg dose-treated eyes at 2 and 3 MPI (*p* = 0.01) and compared to the 8E7 vg dose-treated eyes at 2 MPI (*p* = 0.002) (Figure 6B), indicating that the middle 8E8 vg dose-treated eyes produced the best ERG outcome. These findings suggest that gene therapy with the dose of 8E8 vg can enhance the function of existing rod photoreceptors in treated retinas. However, the beneficial impact of the gene therapy in response to the SCR ERG in eyes treated with the 8E8 vg dose stops at 5 MPI (Figure 6B). The representative waveforms (depicted in Figures 6C,D) demonstrate that among the various doses administered to mice, the medium dose (8E8 vg) was most effective. Compared to WT or heterozygous control mice, the 8E8 vg dose-treated group did not have significant improvements in rod function test at both timepoints (2 and 6 MO) (Figure 6E).

These results suggest that the efficacy of gene augmentation in restoring the loss of function of rod photoreceptors in XLRS is most efficacious at the 8E8 vg dose, although the rescue in rod-dependent retinal function is less robust than the rescue in cone-dependent retinal function.

### 3.6 Subretinal injection of rAAV2-tYF-CB-h*RS1* reduces cyst severity in *Rs1*-KO mice

**Figure 7:**
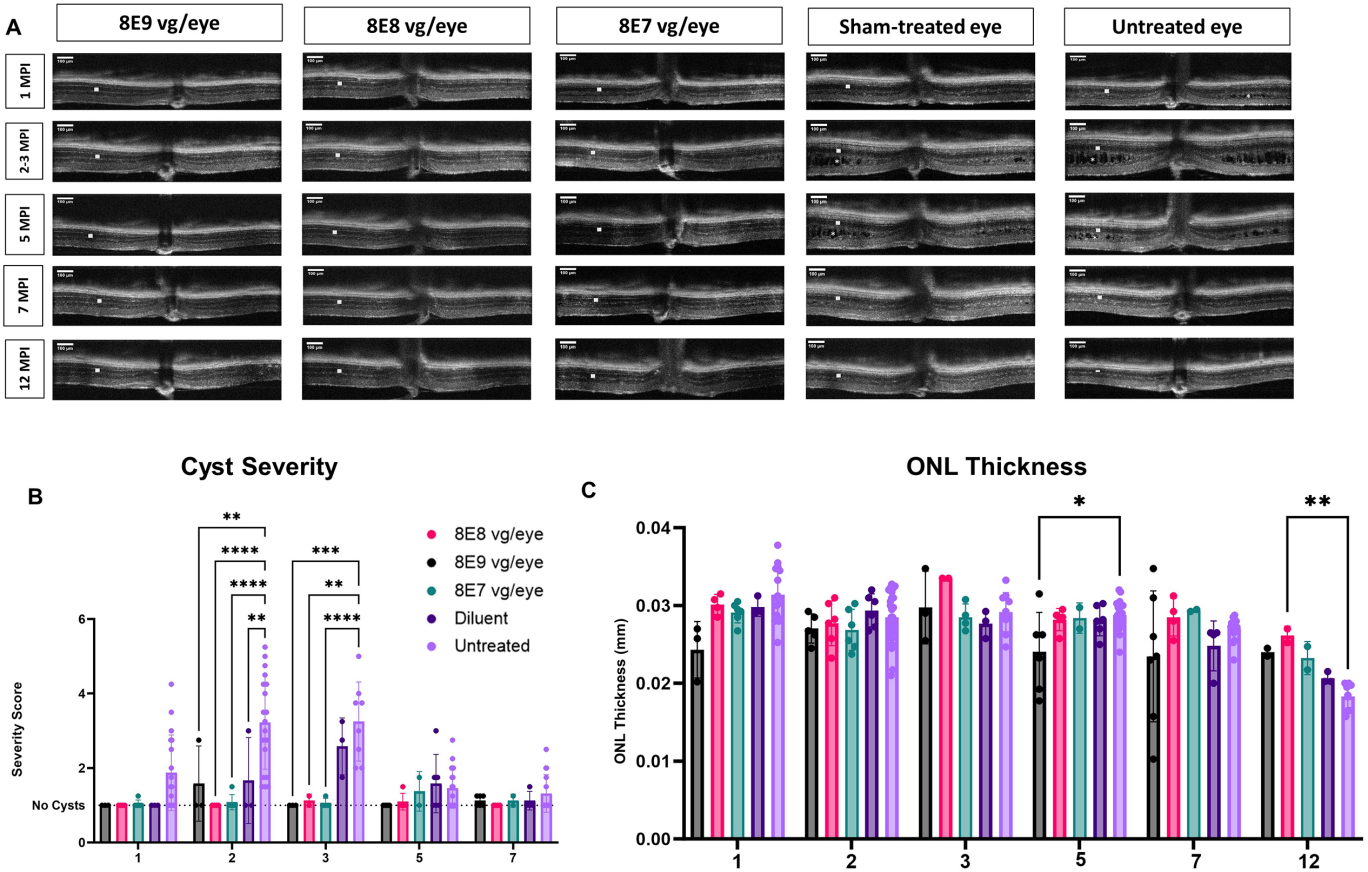
OCT analysis unveils positive outcomes of rAAV2tYF gene therapy over 12 Months. **(A)** OCT images from 1 MPI to 12 MPI showing the cyst reduction and the ONL thickness of all the gene therapy vector treated eyes compared to sham-treated eyes and untreated eyes. Each column of images represents the same mouse, column 3 at 3 MPI timepoint is a different mouse that was treated with the same dose; each column is labeled with the gene therapy dose, the diluent or untreated status. ONL thicknesses are indicated by a white solid bar, and cysts are indicated by white stars on the OCT images. **(B)** Comparison of cyst severity between eyes treated with different doses, diluent treated eyes and untreated contralateral counterparts. Cyst severity was scored on a scale from 1 (least severe, no schisis) to 6 (most severe, schisis size >100 μm) from 1 MPI to 7 MPI. Diluent-treated eyes show a mild reduction in cyst severity, whereas vector-treated eyes show a further reduction in cyst severity compared to the diluent (sham)-treated eyes The results illustrate that eyes treated with the vector demonstrated a more substantial reduction in cyst severity compared to eyes treated with the diluent. **(C)** Comparison of ONL thicknesses between vector treated, sham-treated and untreated contralateral eyes from 1 MPI to 12 MPI. At 12 MPI, eyes treated with 8E8 vg of the vector had significantly thicker ONLs compared to the untreated eyes. *p < 0.05, **p < 0.01, ***p = 0.0001, and ****p < 0.0001. Scale bar, 100 μm.

We performed sequential OCT comparative studies at various time intervals (1, 2, 3, 5, 7 and 12 MPI) to evaluate how the injections of the rAAV2-tYF-CB-h*RS1* viral vector and diluent affect the development of schisis (cysts) and the thickness of the ONL, as illustrated in Figure 7A. At 1 MPI, retinal cysts were apparent in treatment naïve *Rs1-*KO eyes, but not in eyes that received the rAAV2-tYF-CB-h*RS1* viral vector nor the sham treatment (Figure 7A, 1 MPI time point). At 2 MPI (3 months of age), cysts in uninjected eyes had worsened; treatment naïve eyes possessed a significantly higher cyst severity score compared to eyes that received the sham treatment or the rAAV2-tYF-CB-h*RS1* viral vector (8E8 and 8E7 vg: *p* < 0.0001; 8E9: *p* = 0.004; sham treatment: *p* = 0.008) (Figure 7B). It is worth noting that our group has previously reported the beneficial effects of injecting buffer diluent alone on cyst severity in *Rs1*-KO mice (31). At 3 MPI, eyes that received different doses of the rAAV2-tYF-CB-h*RS1* viral vector had less severe cysts compared to eyes that received the sham treatment (Figure 7A). At later time points, OCT images show that cysts naturally resolve over time even in treatment naïve eyes (Figures 7A,B). This observation agrees with Zeng et al. (42) who reported a significant reduction in cavity size between 4 and 8 MO in an *Rs1*-KO mouse model.

To determine whether the injection of the rAAV2-tYF-CB-h*RS1* viral vector had an impact on photoreceptor survival, the thickness of the ONL was measured. From 1 MPI to 12 MPI, treatment naïve eyes in *Rs1-*KO mice experienced a slow photoreceptor degeneration (thickness at 1MPI: 0.031 mm; 7 MPI: 0.027 mm). In early times points (1 MPI to 7 MPI), the ONL thickness showed no notable variations among *Rs1*-KO mice that received 8E8 vg and 8E7 vg doses of rAAV2tYF, the sham treatment, or remained completely untreated (Figures 7A,C). At the experimental end point (12 MPI), mice treated with the 8E8 vg dose had significantly thicker ONL compared to untreated eyes (*p* = 0.006). These results suggest that the 8E8 vg dose, which enables the production of RS1 at near-physiologic levels, is the most efficacious in preventing cell loss. The findings also suggest that the rAAV2tYF vector with the doses of 8E8 and 8E7 vg is well-tolerated by the mice, and no signs of toxicity were observed in the retinal cells following subretinal administration.

### 3.7 Immunohistochemistry confirms 8E8 vg dose cone rescue superiority

**Figure 8:**
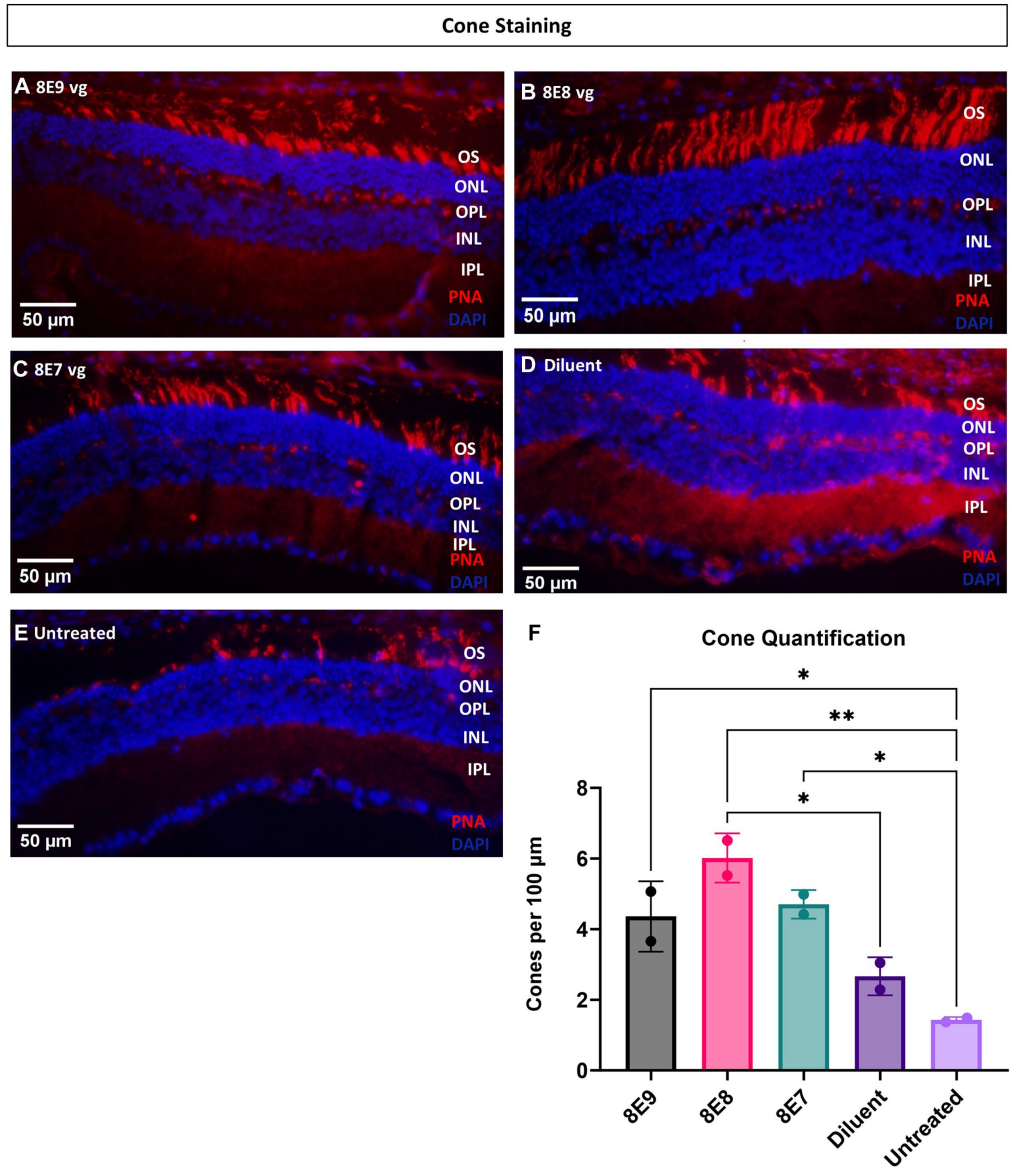
Eyes treated with the 8E8 vg dose exhibit superior cone rescue at 14 months old compared to sham-treated and untreated eyes. **(A–E)** Visualization of cone outer segments using peanut agglutinin. Retinal sections collected from all vector treated eyes, diluent-treated eyes, and untreated *Rs1*-KO eyes at 14 months of age were processed and stained with DAPI (blue) and PNA (red) to visualize cone outer segments. **(F)** Quantification of cone outer segments per 100 μm of the retina indicates that there is a significant preservation of cones in eyes treated with the 8E8 vg dose compared to eyes that received sham treatment and untreated *Rs1*-KO eyes. Eyes treated with all doses of the vector, as well as diluent-treated eyes, had significantly more cones than untreated eyes. Quantification was performed by three individuals masked to treatment groups. OS, outer segment; ONL, outer nuclear layer; OPL, outer plexiform layer; INL, inner nuclear layer; IPL, inner plexiform layer. *p < 0.05, **p < 0.005. Scale bar, 50 μm.

Ninety-eight percent of photoreceptors in mouse retinas are rods, and only 2% of the photoreceptors are cones. In this study, administration of the vector at the 8E8 vg dose enabled robust rescue of cone ERG function over time. Therefore, in addition to using ONL thickness as a measure of cell survival, we also performed quantification of cone photoreceptor cells using IHC. IHC staining was employed to visualize number of cone outer segments in eyes with or without subretinal gene therapy at 14 MO (13 MPI). In this analysis, the outer segments of the cones were stained with biotinylated-PNA (Figures 8A–E). The subsequent quantification process involved imaging and averaging the number of cone photoreceptors to determine the number of cones per 100 μm of the retina. Our results demonstrated a dose-dependent effect on cone survival. Specifically, eyes treated with the 8E8 vg dose exhibited a significantly higher cone survival rate compared to both sham-treated eyes (8E8: 5.83 ± 0.73 cones per 100 μm, *n* = 2; sham-treated eyes: 2.75 ± 0.53 cones per 100 μm, *n* = 2, *p* = 0.018) and untreated eyes (untreated: 1.62 ± 0.11 cones per 100 μm, *n* = 2, *p* = 0.004) (Figure 8F). As expected, the 8E9 vg and 8E7 vg doses treated groups (4.44 ± 0.90 cones per 100 μm, *n* = 2, and 4.63 ± 0.34 cones per 100 μm, *n* = 2 respectively) both of which exhibited significant improvements in cone survival compared to untreated eyes (*p* = 0.02) did not show significance compared to the sham-treated eyes (Figure 8F). These results indicate that the effects seen in eyes that received 8E7 vg or 8E9 vg, although possessing more cones than completely untreated eyes, were not above the beneficial effects seen in buffer injected eyes as previously observed. These findings provide compelling evidence of a dose-dependent rescue effect at the cellular level, reinforcing the therapeutic potential of the rAAV2tYF gene therapy vector with the dose of 8E8 vg in promoting cone survival in the retina.

### 3.8 Incidences of degeneration were observed in the high dose-treated group

**Figure 9:**
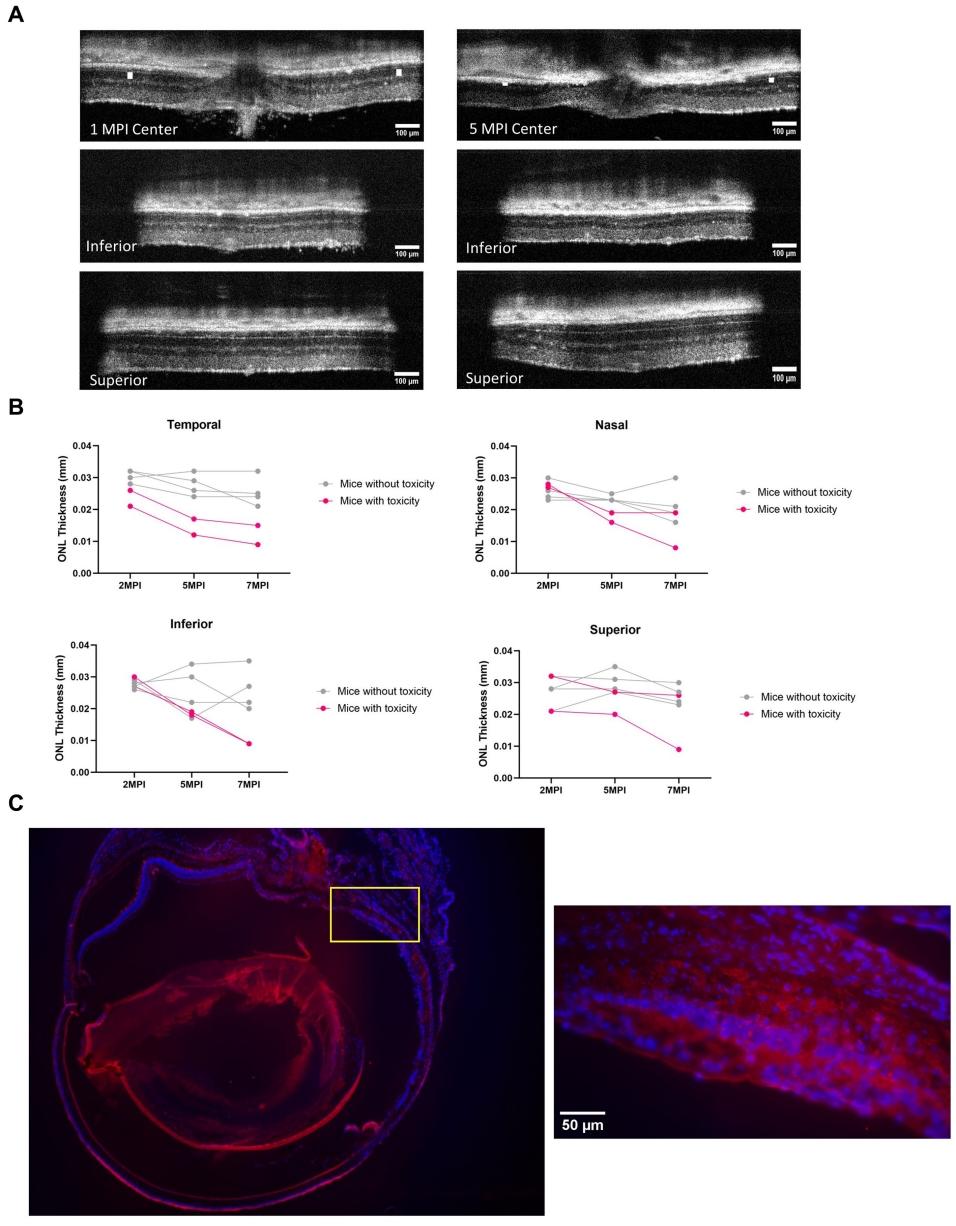
Mice treated with the highest dose (8E9 vg) exhibit signs of toxicity. **(A)** Two out of 6 mice in the 8E9 vg treatment group had localized ONL degeneration in their treated eyes, which was not observed in mice from any other treatment groups nor in sham-treated eyes. OCT images of one mouse (R754) at 1 MPI and 5 MPI reveal ONL degeneration at 5 MPI. The ONL thicknesses are indicated by a white solid bar. Scale bar, 100 µm. **(B)** Analysis of ONL degeneration from 2 MPI to 7 MPI for 6 mice treated with the highest dose (8E9 vg) at four points equidistant to the optic nerve (temporal, nasal, superior, and inferior). Pink lines represent mice with localized ONL degeneration, whereas grey lines represent mice without observed abnormal ONL degeneration. **(C)** Immunohistochemistry (IHC) of a whole eye of the R754 mouse with 4x magnification, stained with DAPI for nucleus visualization and PNA for cone outer segment visualization. The yellow box indicates a 40× magnification of the specified area (inferior to the optic nerve) demonstrating severe loss of the ONL. Scale bar, 50 µm. Center: center scan, Superior: superior side scan, Inferior: inferior side scan.

In both long-term ERG studies and through the quantification of cones, the best rescue was observed in eyes that received the 8E8 vg dose of the rAAV2tYF gene therapy vector. The subretinal injection of the 8E8 vg of rAAV2tYF enabled RS1 protein expression at levels close to those in WT eyes. In contrast, RS1 protein levels in the retina after receiving the 8E9 vg dose exceeded physiological levels. It is important to determine whether there are consequences when RS1 protein expression levels are too high. At 5 MPI, the 8E9 dose treated group had significantly reduced ONL thickness compared to the untreated eyes (*p* < 0.05) (Figure 7C). Further analysis showed two out of 6 mice from two different cohorts of *Rs1*-KO mice treated with the highest dose (8E9 vg) of AAV2tYF had region-specific degeneration exceeding the rates of degeneration seen in other groups as evidenced by OCT images after 5 MPI (Figure 9A). One mouse (R754) had localized photoreceptor degeneration at the temporal and inferior regions of the retina in the treated eye. Another mouse (R816) had accelerated degeneration in all regions of the retina in the treated eye (Figure 9B). In our study, the thicknesses of the ONL were measured at 4 points equidistant to the optic nerve in the retina, and the values reported in Figure 7C represent the average of the 4 measurements in each eye of the mouse. To illustrate the observed region-specific degeneration, measurements at these 4 points in the retina are reported separately for each eye in the 8E9 vg dose group (Figure 9B). These incidences of localized degeneration were not observed in eyes treated with 8E8 vg, 8E7 vg, nor in eyes that received diluent/buffer only (Figure 7C). IHC analysis at 13 MPI (14 MO) of the R754 mouse exhibited severe loss of the photoreceptor layer, particularly in the temporal and inferior points, as corroborated by staining and OCT images (Figures 9A,C). The localized degeneration observed in these mice that received 8E9 vg of the vector suggests a potential link to overexpression toxicity. These findings highlights the importance of optimal dosing in gene therapy treatment for *RS1*.

### 3.9 8E8 vg/eye dose of subretinal gene therapy showed improvements in functional vision in *Rs1*-KO treated mice

To assess the effectiveness of gene therapy on functional vision, we conducted experiments using the VGSA, a method that quantitatively measures rodent functional vision. This test evaluates the visual abilities of mice under various lighting conditions, taking into account both rod and cone-dependent visual pathways. The VGSA is analogous to the multi-luminance mobility test (MLMT) which provides quantitative data on mobility performance and was chosen as an endpoint for the human clinical trials of gene therapy with voretigene neparvovec (now Luxturna^®^) (43, 44). In the VGSA, a mouse is placed in a pool and trained to identify and swim to a platform. The platform’s location is randomized in each trial to prevent the mouse from memorizing its position. After 20 trials, the average time-to-platform (TTP) provides a numerical representation of its functional vision. VGSA approach helps us understand the impact of gene therapy treatment on improving visual function in mice and provides valuable insights for potential treatments in human vision-related conditions (32). We conducted testing under two different lighting conditions: normal room light measuring 13.35 cd/m^2^ and dark in dim red lighting measuring approximately 4.17 × 10^−3^ cd/m^2^. Under the light condition, vision primarily relies on the cone pathway, while in low-light or dark conditions, the rod pathway predominantly supports vision. WT or heterozygous control mice have an average TTP of approximately 3–5 s in both light and dark conditions, and their average TTP remains stable over their life course; this data was previously reported (32). In contrast, we have shown previously that *Rs1-*KO mice have worse functional vision than normal controls at 4–6 months of age in the dark condition (32), and that this swim assay is sensitive enough to detect differences in the rates of visual decline in other inherited retinal diseases (27, 32, 45).

**Figure 10:**
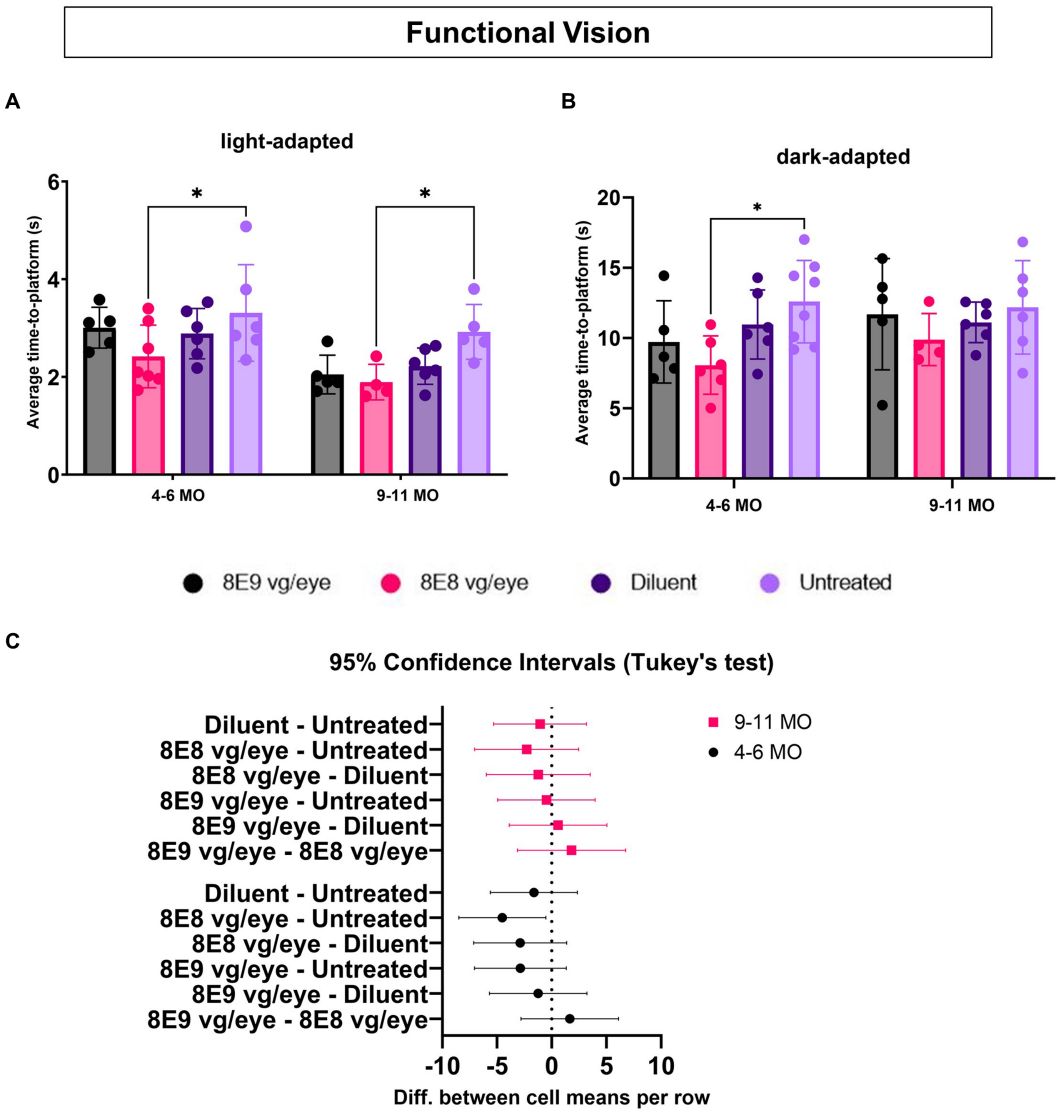
Medium dose (8E8 vg) of subretinal rAAV2tYF gene therapy shows improvement in functional vision. **(A)** Under normal fluorescent ceiling light, *Rs1-*KO mice treated with 8E8 vg dosage of the rAAV2tYF subretinal gene therapy took significantly less time to locate the platform compared to untreated *Rs1-*KO mice but not compared to the sham-treated mice at both time points (4–6 and 9–11 MO). **(B)** In dark testing conditions (dim red lighting), mice treated with the 8E8 vg dose of the gene therapy took significantly less time to locate the platform compared to untreated *Rs1-*KO mice at younger ages but not at older ages. No statistical difference was observed for any of the vector treated eyes compared to the sham-treated eyes. **(C)** Confidence intervals were employed to show the data spread. No statistically significant difference or clear trend towards improvement at older age compared to the untreated eyes was observed. MO: months old, *p < 0.05.

To determine which dose of the subretinal gene therapy (rAAV2tYF) improves the vision of *Rs1*-KO mice, mice that received the 8E9 vg, 8E8 vg doses, the sham-injected mice and untreated *Rs1-*KO mice were tested at 4–6 months of age (3–5 MPI) and then at 9–11 months of age (8–10 MPI) in both light and dark conditions. Untreated *Rs1*-KO mice had an average TTP of 3.30 ± 0.98 s in the light between 4–6 months of age, and 2.92 ± 0.56 s at 9–11 months of age (Figure 10A). Similar data was previously reported (32), showing that *Rs1*-KO mice aged 4–6 MO have a TTP similar to WT controls in bright light. This indicates that functional vision in bright light remained intact. On the other hand, 8E8 vg dose treated *Rs1-*KO mice had an average TTP of 2.42 ± 0.64 s in the light between 4–6 months of age, and 1.89 ± 0.36 s at 9–11 months of age, requiring 26.67% and 35.34% less time to complete the swim experiment than their untreated counterparts at these ages (Figure 10A) with statistical significance (4–6 MO: *p* = 0.04; 9–11 MO: *p* = 0.03). At this time point, 8E8 vg dose treated mice had 15.97 % and 14.86 % less time than their buffer-treated counterparts. Mice treated with 8E9 vg dose or sham-treated eyes did not show any statistical difference compared to the untreated group at both timepoints in the LA conditions. The data obtained from our experiments indicate improvements of the functional vision in the mice treated with the 8E8 vg dose compared to the untreated eyes under LA conditions at both time points (4–6 MO and 9–11 MO). This observation aligns with the improvements seen in the ERG cone function results that were presented earlier.

To determine the effect of subretinal gene therapy on vision in low-light conditions, mediated by rods, the VGSA was performed in the dark and was facilitated by dim red lighting which does not excite the rods. For untreated *Rs1-*KO mice, the average TTP in the dark was 12.58 ± 2.93 s between 4–6 months of age, and 12.17 ± 3.32 s at 9–11 months of age (Figure 10B). Also, it was reported previously that a difference from controls appeared in the *Rs1*-KO DA TTP, indicating that there might be some impairment in their visual capabilities under dark conditions. On the other hand, significant improvements were observed in the TTPs of *Rs1*-KO mice treated with the 8E8 vg dose during dark trials compared to the untreated mice at 4–6 MO. They demonstrated a TTP of 8.06 ± 2.07 s, which represents a 35.87% reduction compared to untreated counterparts (*p* = 0.02). However, at 9–11 MO, the TTP of mice treated with the 8E8 vg dose was 9.88 ± 1.85 s, showing a 18.83% decrease in time compared the untreated mice and 11.08 % decrease in time compared to diluent-treated mice. Despite that, none of the gene therapy treated mice were statistically significant compared to the buffer-treated mice in both light conditions at both time points. We employed confidence intervals to investigate the variability in the data at 9–11 MO, we found no statistically significant difference or clear trend towards improvement. The confidence intervals ranged from −7.049 to 2.460 (Figure 10C). This wide interval indicates that the data points were dispersed on both sides of the mean, with some values showing potential improvement (positive side) and others suggesting a lack of improvement (negative side). In essence, the uncertainty captured by these confidence intervals highlights the complexity of the data at the 9–11 MO time point. This mirror the ERG results for testing the rod function of the group treated with the 8E8 vg dose of the gene therapy, indicating improvements until 3 MPI with a subsequent plateau. The data suggests a temporal window for the therapeutic effects of subretinal gene therapy in *Rs1*-KO mice, particularly evident in behavioral and functional outcomes. These results support the notion that subretinal gene therapy dose of 8E8 provided a benefit in preventing the loss of low-light vision early in the XLRS disease course with more variable rescue in later stages. The advantage displayed by the 8E8 vg dose compared to its counterparts not only underscores its potential as a potent therapeutic agent but also reinforces its potential as a promising candidate for further investigation and clinical development. It is noteworthy that in mice with XLRS, their TTP improved over time, indicating that, similar to humans, mice might learn to adapt and utilize their impaired vision better or it could be due to resolution of cysts with age.

## 4 Discussion

### 4.1 The significance of subretinal gene therapy in contrast to previous intravitreal injection approaches

In two phase I/II trials (NCT02317887 and NCT02416622) for XLRS treatment using AAV8-*RS1* and rAAV2tYF-CB-h*RS1* gene therapy via IVT administration, escalating the dose appeared to correlate with increased inflammation. This inflammatory response wasn’t fully eliminated by steroids, leading to recurrent or chronic inflammation (uveitis) in some cases (18, 19). The observations of gene-therapy-associated uveitis appear to be more frequent among studies using an IVT route of delivery than studies using a subretinal approach (46). Preclinical studies have provided evidence that increases in dosage can lead to an increase in inflammation (5). In nonhuman primates, the IVT delivery of an rAAV2tYF vector expressing green fluorescent protein, processed to enrich for AAV capsids containing the genome (full capsids) or capsids without the genome (empty capsids), demonstrated that ocular inflammation was primarily related to the total amount of capsid delivered and not to transgene expression (47). Although both clinical trials (18, 19) assessed the feasibility of IVT delivery of a gene vector in patients with XLRS, the trials failed to show efficacy of the treatment through the IVT route. This provides motivation/rationale for investigating gene therapy efficacy using subretinal delivery.

### 4.2 Strategic selection of rAAV2tYF-CB-h*RS1* vector for enhanced subretinal gene therapy

The choice of the rAAV2tYF-CB-h*RS1* vector was based on its effective retinal cell targeting, utilizing the enhanced transduction capabilities of the tyrosine-to-phenylalanine mutations in adeno-associated virus serotype 2 (rAAV2tYF) (5). Inducing mutagenesis in surface-exposed tyrosine residues on AAV2 capsids resulted in changes to transduction efficiency, kinetics, and penetration ability during retinal delivery (21). Moreover, the utilization of the chicken β-actin promoter was advantageous in driving robust and enduring gene expression within retinal cells (5), potentially extending therapeutic benefits. Regular AAV2 vectors, though effective in delivering genes to various retinal cells, have limitations in infecting inner retinal cells, with delayed and localized gene expression. Additionally, susceptibility to antibody-mediated immunity poses challenges for readministration (23, 26). This approach presents a promising avenue for precise gene transfer to specific retinal cell subsets.

### 4.3 Insights from *Rs1*-KO mouse model: unveiling the complexities of XLRS pathogenesis and therapeutic prospects

We observed a novel phenomenon, a distinct hyper-normal a-wave response in untreated *Rs1*-KO mice in response to 0.01 dim flash ERG protocol, a characteristic of XLRS not previously reported. This unusual phenomenon suggests that there are compensatory mechanisms at play in the retina. We hypothesize that the disruption of retinal cell organization and the photoreceptor-bipolar cell synaptic structure, attributed to the absence or dysfunction of *RS1*, could trigger adaptive responses. In a normal retina, phototransduction causes photoreceptors to hyperpolarize in response to light, leading to a decrease in glutamate release onto ON bipolar cells. This, in turn, causes ON bipolar cells to depolarize, generating the b-wave of the ERG. The a-wave, which precedes the b-wave, represents the hyperpolarization of photoreceptor cells (48). Studies have proposed that certain aspects of the ERG waveform, specifically the bright flash DA a-wave trough and its immediate recovery, are influenced by depolarizing currents in ON bipolar cells which are driven by cones presumably. It’s suggested that the loss of depolarization in ON bipolar cells could potentially lead to an increased a-wave amplitude in the ERG (48). Because of the mutation in *Rs1* gene and the cellular separation in the inter nuclear layer, we hypothesize that could happen in the 0.01 dim flash ERG test in *Rs1*-KO due to abnormal or disrupted synapses between photoreceptor cells and bipolar cells. This disruption could reduce the efficiency of signal transmission from photoreceptors to bipolar cells which would drive the retina to a negative feedback mechanism to enhance the available signals, which would involve a heightened sensitivity of cone cells to dim light conditions. This would effectively increase the contribution of cone-derived signals to bipolar cells and the downstream retinal layers. Although the precise mechanism requires further clarification, these findings provide insight into the complex relationship between the functionality of retinal cells and synaptic communication. Moreover, the impairments observed in both rod and cone photoreceptor-dependent functions underscore the crucial importance of *RS1* in maintaining robust electrical responses. The reduction in the b/a ratio further mirrored clinical aspects of *RS1*. By investigating subretinal gene therapy intervention during the emergence of disease features, we shed light on potential therapeutic strategies. Our study underscores the importance of *RS1* in maintaining retinal health and reinforces the relevance of the *Rs1*-KO mouse model for exploring XLRS pathogenesis and therapeutic interventions.

### 4.4 Exploring optimal dosage for subretinal gene therapy efficacy

The dose dependent effect was tested in other gene therapy studies for treating XLRS (18, 43). Finding the optimal dose for treating XLRS is critical as previous high doses of rAAV2tYF vector caused inflammation (18). The intriguing dose-response relationship observed in our study provides valuable insights into the optimal therapeutic window for subretinal gene therapy. In our study, the injection of the 8E8 vg dose elicited superior cone-dependent ERGs over the period of 1 year, accompanied by the preservation of cone cells. The rescue effect observed in the 8E8 vg dose group was greater than that observed in eyes receiving a higher or a lower dose. Our western blotting analysis demonstrated that the injection of the 8E8 vg dose enabled RS1 protein expression at levels close to those seen in WT eyes. Even though dose escalation resulted in a dose-dependent increase in RS1 protein levels in the retina, a 10-fold escalation of dose only resulted in a 1.5–2-fold escalation in RS1 expression level. The first possibility is that a 10-fold escalation of dose did not translate to a corresponding increase in terms of the multiplicity of infection. Another possibility is that there is a limit to the capacity of the cells to synthesize and express the protein. This phenomenon could indicate a saturation effect, where the cells reach a maximum threshold in their ability to produce the expressed protein. Our findings confirmed that the second highest dose (8E8 vg) demonstrated superior effects over both lower and higher doses emphasizing the importance of achieving a balance in gene expression. This study found that identifying the optimal dose may be critical when employing a gene delivery approach to treat vision impairment caused by *RS1* mutations. By achieving an optimal range of gene expression, we may observe functional improvements while minimizing the risk of unintended cellular responses.

### 4.5 Integrated approach improves retinal health: insights from ERG, OCT and VGSA analyses

Over 279 pathogenic variants of the *RS1* gene have been linked to XLRS (49). Gene therapy for *RS1* holds significant promise to enhance the lives of these patients. Our study aimed to assess the effectiveness of subretinal gene therapy using rAAV2tYF-CB-h*RS1* vector across different doses (8E9, 8E8, 8E7 vg) in *Rs1*-KO mice. After treating at 3–4 weeks-old, mice were followed to 12 MPI to understand long-term efficacy in comparison to sham-treated and untreated eyes, which was evaluated using multiple experimental modalities, including ERG, OCT, IHC and VGSA.

Our ERG analysis showed although all the gene therapy treated groups showed improvement in the cone function, the second highest dose tested, 8E8 vg, had the highest improvements in the retinal function, sustained and restored cone photoreceptor function until 12 MPI compared to the sham treatment and the untreated eyes, and it was comparable to the WT or heterozygous controls at earlier time points. In addition, the treatment improved the b/a ratio, which is reduced in mice and in patients affected by *RS1* mutations. Our study’s OCT-based insights provide a comprehensive grasp of the treatment’s impact on retinal structure. Notably, with most vector doses, cyst severity decreased, and there were no observed toxic effects except for the highest dose that showed occasional photoreceptor degeneration. It’s worth emphasizing that while the sham treatment did contribute to cyst reduction, the vector treatments exhibited better efficacy in mitigating cyst formation at least at the 3 MPI timepoint. At 14 MO, our OCT results indicate that the 8E8 vg dose preserves the ONL when compared to untreated eyes. This aligns with a previous study (12), which observed significantly thicker ONL in vector-treated eyes compared to untreated eyes at 14 MO time point. This suggests that the viral vector with the 8E8 vg dosage is able to reduce cyst development and rescue the photoreceptor cell layer. Our IHC analysis confirmed the superiority of the 8E8 vg dose on the cellular level in cone rescue at 14 MO. The VGSA showed that treated mice with the medium dose, 8E8 vg, had improvements in the functional vision in the light at younger age, and they took 35.34% less time than untreated *Rs1*-KO mice to complete visual tasks in the light at 9–11 months of age. They also showed improvements in the dark at younger ages (4–6 MO) compared to the untreated naïve mice. These pieces of evidence support the conclusion that subretinal gene augmentation therapy with the dose 8E8 vg for *RS1* improves vision in a *Rs1*-KO animal model.

The reduction of the efficacy of gene therapy in *Rs1*-KO mice over time, while still surpassing untreated eyes by the study’s end, can be attributed to either the elimination of modified cells through immune defense mechanisms, by anti-viral responses (46), or the potential deactivation of therapeutic gene sequences by molecular processes such as episomal silencing (50). While the beneficial effect declines over time, the therapy continues to offer some level of improvement compared to untreated eyes at the study’s conclusion. Investigating the role of the immune system, including measuring antibody levels against the transgene, is the subject of a future study.

In a distinct study conducted by our research team, which investigated the buffer tonicity as a treatment to *Rs1*-KO mice (31), it was observed that mice treated with buffer (whether hypertonic or isotonic) displayed reduced cyst severity and improved cone ERG function in comparison to untreated eyes (31). Nonetheless, the tonicity of the buffer used played a role in the outcomes. While both hypertonic and isotonic buffer treatments were superior to untreated eyes, it was evident that isotonic buffer-treated mice exhibited a lower effect. These results align with our current findings, as the diluent injections in the current study were isotonic and exhibited only marginal improvements over untreated eyes. There is evidence by clinical studies that using topical medications like carbonic anhydrase inhibitors (CAIs) in XLRS patients showed reduced cyst severity and better visual acuity (10, 51). Oral CAIs can also be effective in improving macular morphology and function in XLRS patients, potentially creating favorable conditions for gene therapy.

Our findings emphasize the dose-dependent nature of gene augmentation’s efficacy in preserving rod photoreceptors and restoring cone electrical function in *Rs1*-KO mice. The dosage of gene therapy plays a pivotal role in effectively improving retinal function and functional vision and should be investigated further. These insights contribute to the understanding of gene therapy as a potential treatment strategy for XLRS.

### 4.6 Bridging bench to bedside: translating subretinal gene therapy to clinical practice

The translational implications of our study are twofold. First, the encouraging outcomes observed in our murine model lay a strong foundation for further preclinical investigations, in which the limits of durability can be studied. Second, our findings pave the way for potential clinical trials in humans, suggesting the subretinal route of administration in selected patients may offer better efficacy.

## Data availability statement

The raw data supporting the conclusions of this article will be made available by the authors upon request, without undue reservation.

## Ethics statement

The animal study was approved by Institutional Animal Care and Use Committee (IACUC) protocol #1041421 of the University of Iowa and carried out following the guidelines outlined in the ARVO Statement regarding the use of animals in Ophthalmic and Vision Research. The study was conducted in accordance with the local legislation and institutional requirements.

## Author contributions

SH: Data curation, Formal analysis, Investigation, Methodology, Project administration, Software, Writing – original draft, Writing – review & editing. YH: Data curation, Investigation, Methodology, Supervision, Validation, Writing – review & editing. JT: Data curation, Formal analysis, Methodology, Software, Writing – original draft, Writing – review & editing. EK: Data curation, Writing – review & editing. JV: Data curation, Writing – review & editing. SS: Data curation, Writing – review & editing. AD: Conceptualization, Funding acquisition, Investigation, Methodology, Project administration, Resources, Supervision, Validation, Visualization, Writing – review & editing.
